# Phylogeography of Japanese Encephalitis Virus: Genotype Is Associated with Climate

**DOI:** 10.1371/journal.pntd.0002411

**Published:** 2013-08-29

**Authors:** Amy J. Schuh, Melissa J. Ward, Andrew J. Leigh Brown, Alan D. T. Barrett

**Affiliations:** 1 Center for Biodefense and Emerging Infectious Diseases, University of Texas Medical Branch, Galveston, Texas, United States of America; 2 Center for Tropical Diseases, University of Texas Medical Branch, Galveston, Texas, United States of America; 3 Sealy Center for Vaccine Development, University of Texas Medical Branch, Galveston, Texas, United States of America; 4 Institute for Human Infections and Immunity University of Texas Medical Branch, Galveston, Texas, United States of America; 5 Department of Pathology, University of Texas Medical Branch, Galveston, Texas, United States of America; 6 Institute of Evolutionary Biology, School of Biological Sciences, University of Edinburgh, Edinburgh, Scotland; University of California, Davis, United States of America

## Abstract

The circulation of vector-borne zoonotic viruses is largely determined by the overlap in the geographical distributions of virus-competent vectors and reservoir hosts. What is less clear are the factors influencing the distribution of virus-specific lineages. Japanese encephalitis virus (JEV) is the most important etiologic agent of epidemic encephalitis worldwide, and is primarily maintained between vertebrate reservoir hosts (avian and swine) and culicine mosquitoes. There are five genotypes of JEV: GI-V. In recent years, GI has displaced GIII as the dominant JEV genotype and GV has re-emerged after almost 60 years of undetected virus circulation. JEV is found throughout most of Asia, extending from maritime Siberia in the north to Australia in the south, and as far as Pakistan to the west and Saipan to the east. Transmission of JEV in temperate zones is epidemic with the majority of cases occurring in summer months, while transmission in tropical zones is endemic and occurs year-round at lower rates. To test the hypothesis that viruses circulating in these two geographical zones are genetically distinct, we applied Bayesian phylogeographic, categorical data analysis and phylogeny-trait association test techniques to the largest JEV dataset compiled to date, representing the envelope (E) gene of 487 isolates collected from 12 countries over 75 years. We demonstrated that GIII and the recently emerged GI-b are temperate genotypes likely maintained year-round in northern latitudes, while GI-a and GII are tropical genotypes likely maintained primarily through mosquito-avian and mosquito-swine transmission cycles. This study represents a new paradigm directly linking viral molecular evolution and climate.

## Author Summary

Although Japanese encephalitis virus (JEV) is a major cause of death and disability throughout tropical and temperate Asia, little is known about the evolution, geographical distribution and epidemiology of the five JEV genotypes (genetically distinct groups). To address this gap in our knowledge, we performed a genetic-based geographical analysis using the largest JEV sequence dataset assembled to date, including 487 viral sequences sampled from 12 countries over 75 years. We showed that both the newly and previously dominant genotypes of JEV are associated with temperate climates and are maintained throughout the cold winter months in northern Asia, likely by hibernating mosquitoes (survive throughout the winter), vertical transmission in mosquitoes (female to offspring), cold-blooded vertebrates and/or bats.

## Introduction

Japanese encephalitis virus (JEV) belongs to the JEV serocomplex within the genus *Flavivirus*, family *Flaviviridae*. Recurrent epidemics of summer encephalitis suggestive of JE were recorded in Japan from 1871 onwards and major epidemics occurred in 1924 (6,000 cases, with a 60% case fatality rate), 1929, 1935 and 1937 [1]. The prototype Nakayama strain of JEV was isolated in mice from the brain of a male that died of summer encephalitis in Tokyo, Japan in 1935 [1]. The seasonal occurrence of epidemic encephalitis coupled with the abundance of culicine mosquitoes led to suggestions that JEV was transmitted by mosquito vectors, leading to the subsequent recovery of the virus from rice-paddy breeding *Culex tritaeniorhynchus* mosquitoes in 1938 [2]. A series of ecological studies performed in Japan in the late 1950s established waterbirds as maintenance hosts of the virus, domestic swine as major amplifying hosts, and *Cx. tritaeniorhynchus* as the principal vector between these vertebrate hosts and the incidental, dead-end human host [3], [4], [5], [6], [7], [8], [9], [10], [11], [12].

JEV circulates throughout most of Asia, with the northern limit of virus activity extending north into maritime Siberia. In recent years the geographical distribution of JEV has expanded, reaching east into Saipan in 1990 [13], west into Pakistan in 1992 [14] and south into the Torres Strait between Papua New Guinea and Australia in 1995 [15]. JEV epidemics occur in temperate zones, with the majority of cases occurring in summer or monsoon season months. In contrast, JEV is endemic in tropical regions and transmission occurs year-round at lower rates [16]. Despite the availability of effective vaccines against JEV, the virus is still considered the most important etiologic agent of epidemic encephalitis worldwide, causing an estimated 68,000 cases and a reported 10,000–15,000 deaths annually [17]. Of symptomatic infections, 20–30% are rapidly fatal, 30–50% develop long-term neurologic and/or psychiatric sequelae, and only 20–50% fully resolve the disease [17].

Like other flaviviruses, JEV possesses an 11 kilobase, single-stranded, positive-sense RNA genome containing 5′ and 3′ untranslated regions, and a single open reading frame (ORF) encoding a polyprotein that is co- and post-translationally cleaved by viral and host proteases into three structural proteins: the capsid (C), the precursor of the membrane (prM), and the envelope (E), as well as seven non-structural proteins [18]. The E protein represents the major constituent of the mature virion surface and is the dominant antigen involved in the elicitation of virus neutralizing antibodies [19].

Phylogenetic studies have divided JEV into five genotypes. GI includes isolates collected in northern Australia, northern Cambodia, China, India, Japan, Korea, Laos, Malaysia, Taiwan, Thailand and Vietnam between 1967 and present. GII includes isolates collected sporadically in northern Australia, Indonesia, Korea, Malaysia, Papua New Guinea and southern Thailand between 1951 and 1999. GIII has been the source of annually occurring epidemics of encephalitis and includes isolates collected in China, India, Indonesia, Japan, Korea, Malaysia, Myanmar, Nepal, Philippines, Sri Lanka, the former Soviet Union, Taiwan, Thailand and Vietnam between 1935 and present. GIV includes seven isolates collected in Indonesia between 1980 and 1981 from mosquitoes only. GV includes three isolates collected in Malaysia, China and South Korea between 1952 and 2010. Previous investigations noted GI and GIII viruses were collected mostly in temperate zones, while GII and GIV isolates were collected mostly in tropical zones [20], [21]. However, the statistical significance of this observation has never been tested using a comprehensive dataset of JEV isolates, with information regarding the isolates' genotypes and locations of collection.

The geographical distribution of JEV has expanded in recent years, causing outbreaks of encephalitis in immunologically naïve populations. In addition, the molecular epidemiology of the virus has changed over this period of time. From the isolation of the prototype Nakayama strain of JEV in 1935 until recently, GIII was the most frequently isolated genotype throughout Asia. However, over the past two decades, multiple reports have indicated that GI has displaced GIII as the most frequently isolated virus genotype in a number of Asian countries including China [22], Thailand [23], South Korea [24], Japan [25], Malaysia [26], Vietnam [27], India [28] and Taiwan [29]. Further, following the isolation of the GV Muar isolate [30] in 1952 from an encephalitic patient originating in Malaysia, the genotype remained undetected for almost 60 years until a pool of *Cx. tritaeniorhynchus* collected in the Tibetan Province of China in 2009 yielded the GV XZ0934 isolate [31] and a pool of *Culex bitaeniorhynchus* collected in South Korea in 2010 yielded the GV 10-1827 isolate [32]. A recent evolutionary study utilizing sequence information derived from the ORF of 35 JEV isolates (22 GIII isolates) revealed that JEV originated from its ancestral virus around 1500 [33] and an earlier evolutionary study using 18 genomic JEV sequences (14 GIII) proposed that this evolutionary event occurred in the Indonesia-Malaysia region [34]. Due to the small viral sequence sample sizes, neither of these studies were able to robustly examine the evolution, epidemiology or geographical distribution of the genotypes of JEV. In disagreement with the results of previous studies [33], [34], a recent study utilizing 98 genomic sequences, 76 of which were derived from Chinese virus isolates, estimated that JEV originated from its ancestral virus around 300 AD [35]. This difference was likely due to the slow evolutionary rate estimated for the Chinese JEV study [35] relative to previous JEV evolutionary studies [33], [34], as well other flavivirus evolutionary studies [36], [37], [38], [39]. Prior to the work presented here, no studies have utilized a comprehensive dataset of molecular sequences to examine the phylogeography and epidemiology of the virus genotypes.

Although there is a paucity of ORF sequences of wild-type isolates, extensive sequencing of the phylogenetically-informative E gene of both old and new JEV isolates in recent years has resulted in a large, spatiotemporally distributed collection of viral sequence data. Therefore, we performed a phylogeographic analysis on a dataset consisting of E gene sequence information derived from 487 JEV isolates (largest collection of JEV sequences assembled to date) to address the following key questions: 1) When and where did the virus and its genotypes originate, and what is their geographical range? 2) Is there an association between genotype and climate of virus collection (temperate versus tropical zones)? 3) What amino acid sites within the E protein were involved in the phylogenetic divergence of JEV and were any of these sites subject to diversifying and/or directional selection?

## Materials and Methods

### Dataset creation

All available sequences for the E gene of JEV isolates were retrieved from GenBank in July 2011. The initial JEV E gene dataset was pruned of sequences representing non wild-type virus isolates, duplicate isolates, and isolates absent of information regarding the date and country of collection. The pruned dataset consisted of 489 sequences. The E gene sequences of two JEV isolates (M859/Cambodia/1967/Mosquito and KE-93-83) obtained from the World Reference Center for Emerging Viruses and Arboviruses (WRCEVA) at the University of Texas Medical Branch (UTMB), were determined for analysis in this study utilizing previously described methods [40], [41], [42].

Recombination can invalidate the results of coalescent analyses. Therefore, the nucleotide sequence alignment file was analyzed for potential recombination events using RDP [43], GENECONV [44], Chimaera [45], MaxChi [46] and Bootscan [47] methods implemented in RDP3 v Beta 41 [48]. Common program settings were to perceive sequences as linear, require phylogenetic evidence, refine breakpoints and check alignment consistency, while all method-specific program settings remained at their default values. The highest acceptable p-value was set at 0.05, after considering Bonferroni correction for multiple comparisons. Potential recombination events were those that were identified by at least two methods. The breakpoint positions and recombinant sequence inferred for the potential recombination events were manually confirmed using the phylogenetic and recombination signal analysis features in RDP3. The K82P01 and K91P55 sequences were confirmed as recombinants (Table S1). These two isolates were not available from the WRCEVA at UTMB to re-sequence; therefore, the two corresponding sequences were removed from the dataset, leaving a final dataset of 487 sequences.

To make an initial identification of the genotype of the JEV E gene sequences, neighbor-joining (NJ) and maximum-likelihood (ML) phylogenies were generated using SeaView v 4.2.12 [49] and PhyML v 3.0 on the South of France bioinformatics platform [50], respectively.

The final dataset of 487 JEV E gene sequences included information regarding the year, host and country of collection of the corresponding virus isolates. Sequences derived from isolates collected north of the Tropic of Cancer (23.5°N) were classified as temperate, while sequences derived from isolates collected south of the Tropic of Cancer were classified as tropical. The climate corresponding to five Taiwanese sequences could not be ascertained and therefore these sequences were not included in the climate phylogeographic analysis described below.

### Construction of time-scaled phylogenies and phylogeographic analyses

To estimate the date and location of the most recent common ancestor (MRCA) of the five genotypes and the overall rate of molecular evolution, time-scaled Bayesian phylogenies (country and climate) were inferred from the JEV E gene sequence dataset using a Bayesian Markov Chain Monte Carlo (MCMC) method implemented in BEAST v 1.6.1 [51].

An SDR06 nucleotide substitution model [52], a relaxed-uncorrelated exponential molecular clock and a piecewise constant Bayesian skyline demographic model with 20 coalescent-interval groups [53] were used in all analyses. The relaxed-uncorrelated exponential molecular clock was found to best-fit the data when Bayes factor (BF) values were calculated (Tracer v 1.5.1) [54] to evaluate the relative fit of strict and relaxed molecular clock models to the data by determining the natural logarithm of the ratio of the marginal likelihoods of the competing models [55]. The good fit of this relaxed clock model to the data has recently been shown to be an artifact of the harmonic mean estimator [56]. However, preliminary analyses showed that the selection of a particular relaxed molecular clock model had little effect on the results.

To infer the probable geographic origin of the MRCA of the genotypes of JEV, the BEAST input files (country and climate) created in BEAUti v 1.6.1 [51] were edited to include the Bayesian stochastic search variable selection procedure [57].

The Bioportal at the University of Oslo [58] was used to execute the MCMC analyses for 600 million generations. This was achieved by using LogCombiner v 1.6.1 [51] to compile 12 independent runs of 50 million generations (sampled every 1,000^th^ state) to attain convergence, which was assessed by examining the trace and effective sample size statistics for each model parameter in Tracer v 1.5 [54]. TreeAnnotator v 1.6.1 [51] was used to summarize the posterior tree distribution and annotate both country and climate maximum clade credibility (MCC) phylogenies, which were viewed in FigTree v 1.3.1 [59]. Each of the nodes of the Bayesian MCC phylogenies were annotated with posterior probability (PP) values, estimated median dates of the MRCA with corresponding 95% HPD values, and state PP values for each plausible geographic location of origin (country and climate). In addition, BOA v 1.15 [60] implemented in R v 2.15.1 [61] was used to calculate a 50% HPD interval for the date of the root of the phylogeny.

Maps showing the distributions of sequences according to sampling location (country and climate) were created using GIMP v 2.6.12 from a blank map of Asia.

### Distribution of JEV isolates according to genotype and climate

To test the null hypothesis of no association between genotype and climate, a Fisher's exact test was performed at α = 0.05 (IBM SPSS Statistics v 20). Post-hoc analyses were then performed to determine which cell(s) in the table of genotype versus climate contributed the most to the statistically significant Fisher's exact test. Adjusted standardized residuals (z-scores) were calculated and the Bonferroni method was used to correct for multiple comparisons. The adjusted standardized residual values were then compared against the critical z-value (±1.96) for α = 0.05 (IBM SPSS Statistics v 20). Only GI-a, GI-b, GII and GIII were considered in these analyses, as the dataset included only three sequences each for GIV and GV.

The null hypothesis of no phylogeny-trait association was further evaluated at α = 0.05 using the association index (AI), parsimony score (PS), unique fraction (UniFrac), nearest taxa (NT), net relatedness (NR), phylogenetic diversity (PD) and maximum exclusive single-state clade size (MC) statistics calculated from the posterior set of trees generated by BEAST in Befi-BaTS v 0.1.1 [62].

### Identification of genotype-defining substitutions and molecular adaptation analyses

Nonsynonymous substitutions involved in the phylogenetic divergence of the five genotypes of JEV were identified within the E protein alignment. The E gene alignment was evaluated for statistically significant evidence of positive selection (ratio of nonsynonymous to synonymous nucleotide substitutions [d_N_/d_S_] >1; p<0.05) using the single-likelihood ancestor counting (SLAC), fixed effects likelihood (FEL) and internal FEL (IFEL) methods [63] available on the Datamonkey webserver [64]. All analyses of positive selection utilized a NJ phylogeny and the reversible nucleotide substitution model. Evidence of directional selection within the E protein alignment was evaluated using the directional evolution of protein sequences (DEPS) [65] method implemented in HyPhy v 2.0 [66]. The DEPS method utilized a Bayesian phylogeny and the Jones, Taylor, Thorton amino acid substitution model to assess for the presence of statistically significant shifts in amino acid residue frequencies (p<0.05) and/or a statistically significant large number of substitutions toward a particular residue (BF>100).

## Results

### Descriptive analysis of the JEV dataset

Of the 487 isolates in the JEV dataset (Table S2), the majority belonged to GI-b and GIII, the most common viral host was the mosquito, the most frequent decade of isolation was the 2000s, the most common country of virus isolation was Japan and the majority of isolates were collected in temperate climates (Table 1).

**Table 1:** Descriptive analysis of the JEV sequence dataset.

		n	% of total
**Genotype**	GI-a	15	3.08
	GI-b	219	44.97
	GII	28	5.75
	GIII	219	44.97
	GIV	3	0.62
	GV	3	0.62
	Total	487	100.00
**Host**	Equid	4	0.82
	Human	67	13.76
	Midge	3	0.62
	Mosquito	282	57.91
	Swine	98	20.12
	Unknown	33	6.78
	Total	487	100.00
**Decade**	1930s	2	0.41
	1940s	5	1.03
	1950s	19	3.90
	1960s	11	2.26
	1970s	48	9.86
	1980s	62	12.73
	1990s	33	6.78
	2000s	307	63.04
	Total	487	100.00
**Origin**	Australia	5	1.03
	Cambodia	1	0.21
	China	126	25.87
	India	8	1.64
	Indonesia	26	5.34
	Japan	207	42.51
	Korea	34	6.98
	Malaysia	3	0.62
	Sri Lanka	1	0.21
	Taiwan	44	9.03
	Thailand	14	2.87
	Vietnam	18	3.70
	Total	487	100.00
**Climate**	Temperate	406	83.37
	Tropical	76	15.61
	Unknown	5	1.03
	Total	487	100.00

### Rate of molecular evolution

The overall median evolutionary rates estimated from the JEV E gene country and climate datasets were 5.33×10^−4^ (95% HPD: 3.92×10^−4^, 6.52×10^−4^) and 5.51×10^−4^ (95% HPD: 4.24×10^−4^, 6.67×10^−4^) substitutions/site/year, respectively. These estimates are slightly higher and the 95% HPD intervals are slightly wider compared to estimates previously obtained from a dataset of 35 JEV ORF sequences (mean: 4.35×10^−4^ substitutions/site/year, 95% HPD: 3.49×10^−4^, 5.30×10^−4^) [33]. This variation was likely due to the fact that more temporal signal can be extracted from longer alignments.

### Spatiotemporal chronology of the evolution of JEV

**Figure 1:**
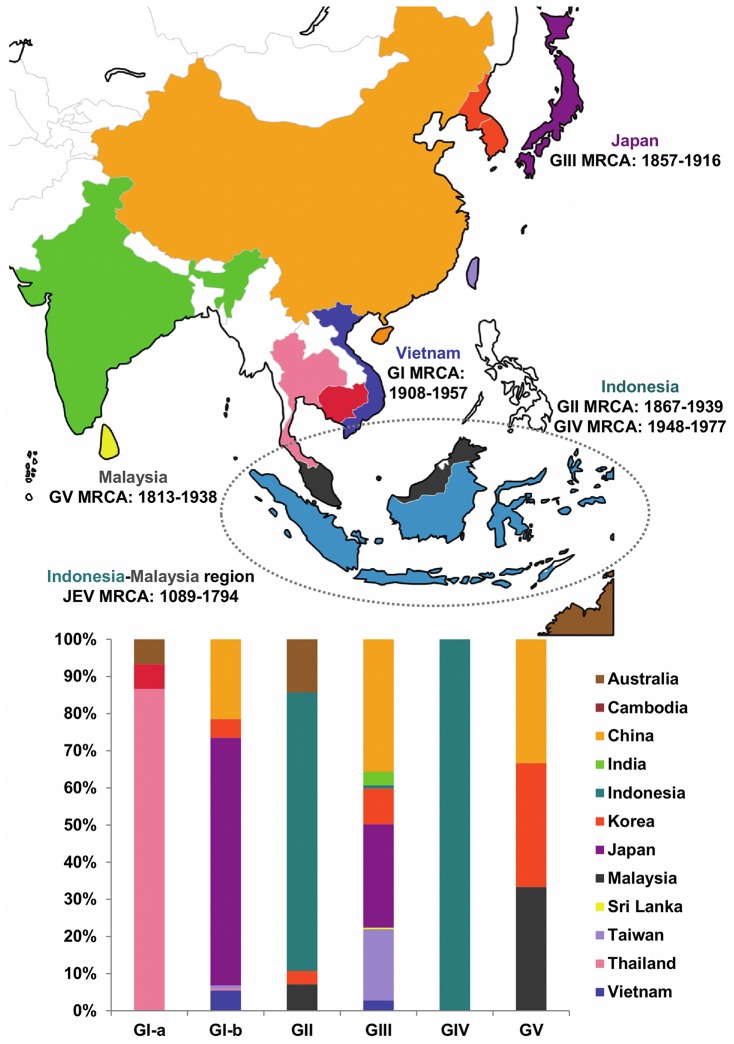
Geographical distribution of the JEV sequences included in this study according to country of collection. The coloring of the chart corresponds to the map and shows the relative proportions of viral sequences sampled from each country according to genotype. Of note, GI viruses have also been isolated in India, Laos and Malaysia, GII viruses have also been isolated in Papua New Guinea and Thailand, and GIII viruses have also been isolated in Malaysia, Myanmar, Nepal, the Philippines, the former Soviet Union and Thailand. These viruses were not included in this study, as the E gene of these viruses has not been sequenced. The estimated dates (95% HPD interval) and locations for the MRCA of JEV, as well as its five genotypes are also shown.

**Figure 2:**
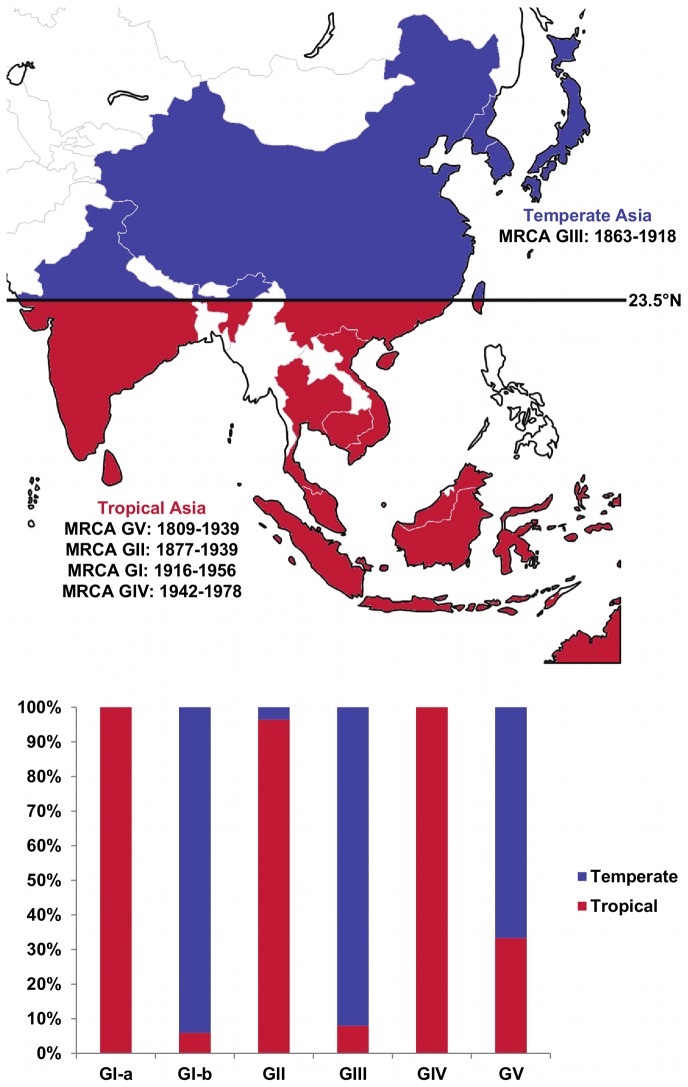
Geographical distribution of the JEV sequences included in this study according to climate of collection. The coloring of the chart corresponds to the map and shows the relative proportions of viral sequences sampled from each climate according to genotype. The estimated dates (95% HPD interval) and location for the MRCA of JEV, as well as its five genotypes are also shown.

**Figure 3:**
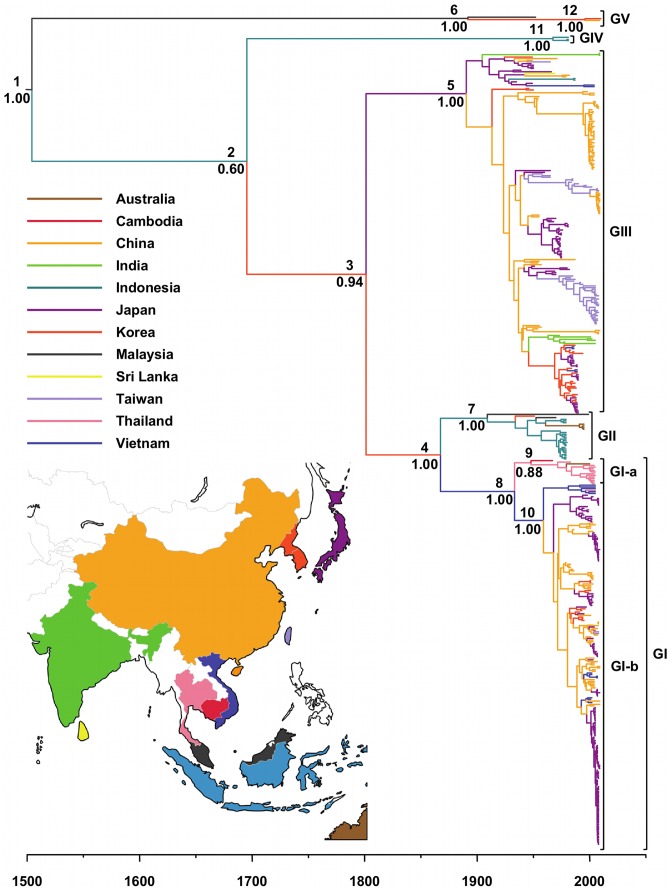
Country Bayesian MCC phylogeny of the JEV sequences. GI-V are represented to the right of the tree. Branch tips correspond to the date of collection of each of the virus isolates from which JEV sequence information was derived. Branch lengths correspond to lengths of time (in years), as measured by the scale underneath the tree. Terminal branches are colored according to the sampling location of the taxon at the tip, while internal braches are colored according to the most probable location of their child node. The branch colors correspond to those used in the map and legend. The numbers to the upper-left of the nodes correspond to the country phylogeographic analysis date presented in Table 2 and the numbers to the lower-left of the nodes are posterior probability values.

**Figure 4:**
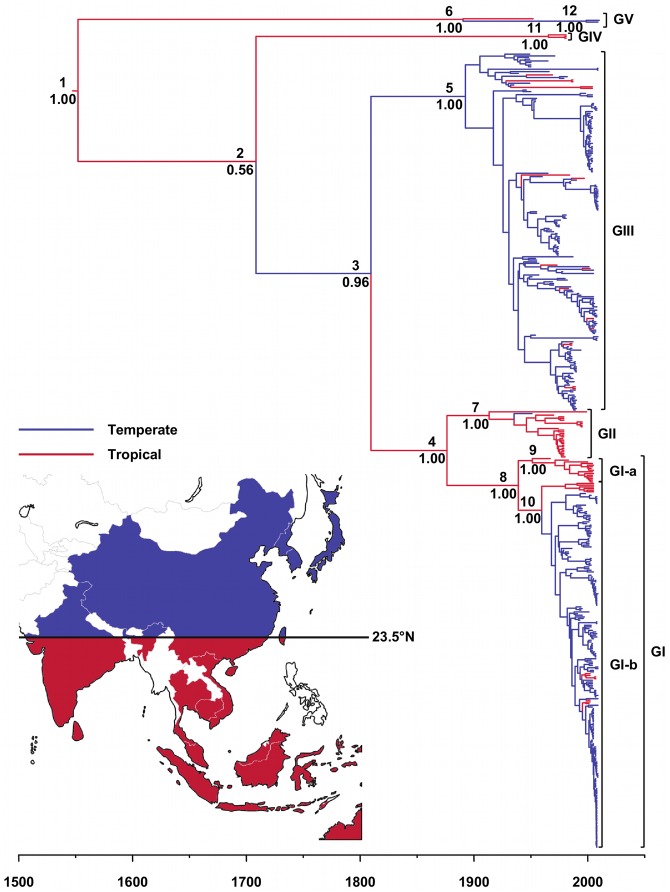
Climate Bayesian MCC phylogeny of the JEV sequences. GI-V are represented to the right of the tree. Branch tips correspond to the date of collection of each of the virus isolates from which JEV sequence information was derived. Branch lengths correspond to lengths of time (in years), as measured by the scale underneath the tree. Terminal branches are colored according to the sampling location of the taxon at the tip, while internal braches are colored according to the most probable location of their child node. The branch colors correspond to those used in the map and legend. The numbers to the upper-left of the nodes correspond to the country phylogeographic analysis date presented in Table 3 and the numbers to the lower-left of the nodes are posterior probability values.

Figures 1 and 2 show the geographical distribution of the JEV sequences included in this study according to the country and climate of collection, respectively. Country and climate Bayesian MCC phylogenies are shown in Figures 3 and 4, respectively. As expected, the topologies of the BEAST phylogenies are supported by the NJ and ML phylogenies (Figures S1 and S2), and are similar to recently published phylogenies generated from both ORF and E gene sequence information for GI-V of the virus [31], [32], [33]. All four of the phylogenies inferred in this study support the division of GI into two clusters, GI-a and GI-b, where the GI-a clade consists of 15 isolates sampled in Cambodia, Thailand and Australia between 1967 and 2005 and GI-b includes 219 isolates sampled from Vietnam, Thailand, Japan, Korea, China and Taiwan between 1979 and 2009. Estimated dates of the MRCA and state PP values in support of each of the 12 countries are presented in Table 2 for the key nodes within the country Bayesian MCC phylogeny (Figure 3), and estimated dates of the MRCA and state PP values in support of tropical and temperate climates of divergence are presented in Table 3 for the key nodes within the climate Bayesian MCC phylogeny (Figure 4).

**Table 2:** Country phylogeographic analysis.

			State PP											
Node	Group	Date of the MRCA (95% HPD)	Australia	Cambodia	China	India	Indonesia	Japan	Korea	Malaysia	Sri Lanka	Taiwan	Thailand	Vietnam
1	JEV	1553 (1089, 1794)	0.08	0.05	0.07	0.03	0.18	0.09	0.10	0.19	0.05	0.03	0.06	0.07
2	GIV+GIII+GII+GI	1709 (1516, 1844)	0.06	0.03	0.08	0.02	0.21	0.11	0.14	0.16	0.04	0.02	0.05	0.08
3	GIII+GII+GI	1811 (1701, 1880)	0.04	0.02	0.13	0.01	0.14	0.18	0.20	0.10	0.02	0.01	0.04	0.11
4	GII+GI	1875 (1799, 1920)	0.07	0.02	0.05	0.00	0.22	0.07	0.24	0.12	0.02	0.00	0.05	0.14
5	GIII	1894 (1857, 1916)	0.00	0.00	0.26	0.00	0.00	0.57	0.16	0.00	0.00	0.00	0.00	0.01
6	GV	1902 (1813, 1938)	0.06	0.01	0.03	0.01	0.22	0.04	0.12	0.40	0.03	0.01	0.03	0.04
7	GII	1913 (1867, 1939)	0.05	0.01	0.01	0.00	0.40	0.03	0.18	0.22	0.02	0.00	0.01	0.07
8	GI	1936 (1908, 1957)	0.06	0.06	0.05	0.00	0.03	0.02	0.09	0.02	0.01	0.00	0.22	0.44
9	GI-a	1949 (1927, 1962)	0.08	0.18	0.01	0.00	0.01	0.00	0.03	0.02	0.01	0.00	0.43	0.23
10	GI-b	1961 (1941, 1971)	0.00	0.00	0.27	0.00	0.00	0.04	0.12	0.00	0.00	0.00	0.01	0.56
11	GIV	1971 (1948, 1977)	0.00	0.00	0.00	0.00	0.98	0.00	0.00	0.02	0.00	0.00	0.00	0.00
12	XZ0934/10-1827	1997 (1982, 2006)	0.00	0.00	0.28	0.00	0.00	0.15	0.51	0.00	0.00	0.00	0.00	0.05

**Table 3:** Climate phylogeographic analysis.

			State PP	
Node	Group	Date of the MRCA (95% HPD)	Temperate	Tropical
1	JEV	1590 (1207, 1791)	0.47	0.53
2	GIV+GIII+GII+GI	1723 (1540, 1839)	0.48	0.52
3	GIII+GII+GI	1822 (1704, 1884)	0.53	0.47
4	GII+GI	1883 (1818, 1923)	0.27	0.73
5	GIII	1896 (1863, 1918)	0.97	0.03
6	GV	1902 (1809, 1939)	0.35	0.65
7	GII	1917 (1877, 1939)	0.23	0.77
8	GI	1941 (1916, 1956)	0.13	0.87
9	GI-a	1953 (1936, 1964)	0.03	0.97
10	GI-b	1961 (1972, 1944)	0.33	0.67
11	GIV	1970 (1942, 1978)	0.01	0.99
12	XZ0934/10-1827	2000 (1986, 2007)	0.99	0.01

The country Bayesian MCC phylogeny (Figure 3) and the country map (Figure 1) show that GV includes three isolates sampled in China, South Korea and Malaysia between 1952 and 2010, GIV includes three isolates sampled in Indonesia between 1980 and 1981, GIII includes 234 isolates sampled in China, India, Indonesia, Japan, Korea, Sri Lanka, Taiwan and Vietnam between 1935 and 2009, GII includes 28 isolates sampled in Australia, Indonesia, Korea and Malaysia between 1951 and 1999, GI-a includes 15 isolates sampled in Cambodia, Thailand and Australia between 1967 and 2005, and GI-b includes 219 isolates sampled from Vietnam, Thailand, Japan, Korea, China and Taiwan between 1979 and 2009.

Phylogeographic analysis estimated that the date of the MRCA of JEV lies between 1506 and 1704 with a posterior probability of 50%, and between 1022 and 1800 with a posterior probability of 95% (median: 1553, 50% HPD: 1506, 1704; 95% HPD: 1022, 1800). These estimates are consistent with those recently inferred using a dataset of 35 JEV ORF sequences (mean: 1559; 95% HPD: 1509, 1635 [33]. The increased width of the 95% HPD intervals for the date of the MRCA of JEV for the E gene sequence dataset compared to the ORF gene sequence dataset can again be attributed to the fact that more temporal signal can be extracted from longer alignments. In agreement with previous suggestions regarding the origin of JEV [34], the root (MRCA of JEV) state PP values for all locations range between 0.03 and 0.19, with the highest state posterior probability values corresponding to Malaysia and Indonesia (0.19 and 0.18, respectively).

Of the five genotypes of JEV, the MRCA of GIII occurred earliest in time (median: 1894; 95% HPD: 1857, 1916) possibly in Japan (state PP: 0.57), followed by the MRCA of GV (median: 1902; 95% HPD: 1813, 1938) possibly in Malaysia (state PP: 0.40), the MRCA of GII (median: 1913; 95% HPD: 1867, 1939) possibly in Indonesia (state PP: 0.40), the MRCA of GI (median: 1936; 95% HPD: 1908, 1957) possibly in Vietnam (state PP: 0.44) and, most recently, the MRCA of GIV (median: 1971; 95% HPD: 1948, 1977) possibly in Indonesia (state PP: 0.98) (Figure 3, Table 2).

Within GI, the MRCA of GI-a occurred first (median: 1949; 95% HPD: 1927, 1962) possibly in Thailand (state PP: 0.43), followed by the MRCA of GI-b (median: 1961; 95% HPD: 1941, 1971) possibly in Vietnam (state PP: 0.56) (Figure 3, Table 2).

The MRCA of the recently emerged GV isolates (XZ0934 [China, 2009] and 10-1827 [South Korea, 2010]; node PP: 1.00) occurred recently (median: 1997; 95% HPD: 1982, 2006) possibly in Korea (state PP: 0.51) (Figure 3, Table 2).

The most striking observation from the climate Bayesian MCC phylogeny (Figure 4) and the climate map (Figure 2) is that GV includes isolates sampled from temperate and tropical locations, GIV includes isolates sampled from only tropical locations, GIII and GI-b include isolates sampled primarily from temperate locations, and GII and GI-a include isolates sampled primarily from tropical locations. The posterior probabilities for a tropical or temperate climate at the root of the tree were approximately equal (tropical state PP: 0.53, temperate state PP: 0.47) (Figure 4, Table 3). The MRCA of GIII (state PP: 0.97) and the recently emerged GV isolates (state PP: 0.99) was most likely in temperate Asia, while the MRCA of GV (state PP: 0.65), GII (state PP: 0.77), GI (state PP: 0.87), GI-a (state PP: 0.97), GI-b (state PP: 0.67) and GIV (state PP: 0.99) was most likely in tropical Asia (Figure 4, Table 3).

### Relationship between genotype and climate

A Fisher's exact test was used to statistically evaluate the observed relationship between genotype and climate. Based on α = 0.05, we rejected the null hypothesis of no genotype-climate association and concluded that there was a statistically significant relationship between genotype and climate (Fisher's exact test: 173.48; exact two-sided p-value: 0.000). Post-hoc analysis revealed that GIII included significantly more isolates sampled from temperate climates than expected (adjusted standardized residual: 4.0), GII included significantly more isolates sampled from tropical climates than expected (adjusted standardized residual: 12.4), GI-a included significantly more isolates sampled from tropical climates than expected (adjusted standardized residual: 9.3) and GI-b included significantly more isolates sampled from temperate climates than expected (adjusted standardized residual: 5.2). The phylogeny-trait association test of genotype-climate phylogenetic structure also failed to reject the null hypothesis of no association between genotype and climate (Table S3); thereby, providing further evidence that JEV genotype is associated with climate.

### A single genotype-defining substitution is associated with the adaptive evolution of JEV

Forty-four genotype-defining nonsynonymous substitutions were identified within the E protein (Table S4), 36 of which were GV-specific (13 non-conservative). Selection analyses were then performed to determine if any of these genotype-defining nonsynonymous substitutions might have played a role in the adaptation of the viral genotypes to their respective environments. No positively selected sites were identified using the SLAC, FEL and IFEL methods. However, the DEPS method revealed elevated substitution rates towards seven residues (Table S5) and thirteen sites were identified to be involved in this directional evolution (Table S6). One of these directionally selected sites (129; preferred residue: M) corresponded to the site of a genotype-defining nonsynonymous substitution (129 [GV: I, GIV: T, GIII: T, GII: T, GI: M]).

## Discussion

### JEV originated in the Indonesia-Malaysia region around the sixteenth century

In accordance with previous analyses, we estimated that JEV originated in the Indonesia-Malaysia region [34] around the 16^th^ century [33]. However, as expected for a node so far back in time, the posterior probability values in support of the origination of JEV in the Indonesia-Malaysia region were low. Nevertheless, as emphasized previously, all virus genotypes have been found in the Indonesia-Malaysia region and large epidemics suggestive of JEV have never been reported to occur in this region [34]. These lines of evidence are consistent with the virus having evolved in the Indonesia-Malaysia region [34]. Interestingly, based on the results of an amino acid signature analysis, others have suggested that Asian JEV and Australian Murray Valley encephalitis virus may have evolved from a virus related to the African Usutu virus in the Southeast Asia-Australasia region [67].

### GIII is significantly associated with temperate climates

Phylogeographic analysis estimated that GIII evolved in temperate Asia (Japan) around the late 19^th^ century. These estimates are coincident with the first reported summer epidemics of encephalitis suggestive of JE, which occurred in 1871 in Japan [1]. Following the isolation of the prototype Nakayama strain of JEV (a GIII virus) from Japan in 1935, GIII has been found throughout most of Asia including China, India, Indonesia, Korea, Japan, Sri Lanka, Taiwan and Vietnam.

Statistical analyses indicated that GIII did indeed include significantly more temperate isolates than expected under the null hypothesis of no association between genotype and climate. The paucity of GIII viruses sampled from tropical regions and the genetic relatedness of GIII viruses sampled years apart suggests that the annual re-introduction of GIII viruses from tropical regions to temperate regions by migratory birds or wind-blow mosquitoes does not seem to play a large role in the epidemiology of GIII. Rather, GIII is most likely maintained year-to-year by hibernating mosquitoes, vertical transmission in mosquitoes, poikilothermic vertebrates and/or bats. In support of this hypothesis, JEV has been isolated from overwintering *Culex sp.* in temperate Asia [68], virus transmission by *Culex spp.* was shown following experimental hibernation [69], and vertical transmission of JEV in *Culex spp.* and *Armigeres sp.* has been experimentally demonstrated [70], [71]. Furthermore, antibody to JEV has been detected in several poikilothermic vertebrates [71], [72], [73], [74], experimentally JEV-infected lizards were able to maintain the virus throughout the winter [72], and experimental transmission of JEV has been shown from infected mosquitoes to uninfected lizards and from infected lizards to mice through mosquitoes [72]. In temperate Asia, JEV has been isolated from several bat species [73], [74] and JEV-infected bats subjected to experimental hibernation were able to maintain their viremias for over 100 days [75].

### GV recently re-emerged after 60 years of undetected virus circulation

The MRCA of the three GV sequences was estimated to have existed in the early 20^th^ century. Although the node states were largely influenced by the small number of GV sequences (n = 3), the mass of posterior probability supporting the location of GV evolution corresponds to tropical Asia, specifically Malaysia. In Malaysia, JEV was first described in the 1940s when an outbreak occurred during the Second World War among British prisoners of war [76]. It is possible that GV may have circulated undetected in tropical Asia for much longer, causing only sporadic cases of encephalitis that were mistaken for cerebral malaria or other encephalitic diseases.

Surprisingly, after almost 60 years of undetected virus circulation, a pool of *Cx. tritaeniorhynchus* collected in the Tibetan Province of China in 2009 yielded the GV XZ0934 isolate [31] and a pool of *Culex bitaeniorhynchus* collected in South Korea in 2010 yielded the GV 10-1827 isolate [32]. The MRCA of the XZ0934 and 10-1827 isolates was estimated to have occurred sometime within the last 27 years in temperate Asia. Interestingly, despite surveillance neither JEV nor *Cx. tritaeniorhynchus* had been detected in Tibet prior to 2009 [77], suggesting that GV of JEV may have entered Tibet shortly before it was initially isolated in 2009. It is possible that GV arrived in Tibet via JEV-infected migratory birds or perhaps by wind-blown mosquitoes.

The three GV viruses shared 36 genotype-defining nonsynonymous substitutions within the E protein, 13 of which were non-conservative. This is consistent with the Muar strain's distinct serological classification based on its reactivity with a set of monoclonal antibodies [78]. None of the GV isolates have been characterized using polyclonal antibodies derived from other members of the JEV serocomplex. Such studies may provide interesting information regarding the antigenic relationships between this ancestral JEV genotype and other closely related viruses, such as Murray Valley encephalitis virus and Usutu virus.

### GII is significantly associated with tropical climates

We estimated that GII evolved in tropical Asia around the early 20^th^ century. The Bennett isolate, made in Korea *circa* 1951, represents the only example of a GII virus collected outside of tropical Asia [40]. As extensive surveillance for JEV has been performed in temperate Asia, this single genotype isolation event likely coincided with a GII epidemic focus that quickly died off [40]. Therefore, as predicted, statistical analyses demonstrated that GII included significantly more isolates sampled from temperate regions than expected.

### GI-b, a temperate genotype, has recently displaced GIII as the dominant viral genotype of JEV throughout Asia

It was estimated that GI, GI-a and GI-b all emerged in tropical Asia around the mid 20^th^ century. Statistical analyses demonstrated that GI-a included significantly more isolates sampled from tropical regions, and GI-b included significantly more isolates sampled from temperate regions, than expected under the null hypothesis of no association between climate and genotype. Like GIII, GI-b may be maintained in temperate Asia throughout the winter months in hibernating mosquitoes, vertical transmission in mosquitoes, poikilothermic vertebrates, and/or bats. This suggests that the spread and establishment of GI-b throughout Asia may have been due to its ability to efficiently overwinter in temperate Asia.

The phylogenetic divergence of GI is defined by a non-conservative threonine to methionine substitution at site 129 of the E protein. This GI-defining substitution was also found to be under directional selection. Although substitutions at this site within the E protein of JEV have not yet been associated with phenotypic alterations, this substitution alone or in combination with substitutions in other regions of the genome may have provided a phenotypic advantage to GI viruses that led to the spread and establishment of this genotype throughout Asia.

### GIV is geographically confined to Indonesia

The MRCA of the three GIV sequences was estimated to have existed in the late 20^th^ century. GIV includes seven isolates (only three of these include E gene sequence information) collected from mosquitoes only on three islands encompassing the Indonesian archipelago between 1980 and 1981. The reasons why GIV appears to be confined to Indonesia are unknown, but could be due to a number of reasons. For example, there could be a narrow host/vector range for GIV, the vector competence of *Cx. tritaeniorhynchus* for GIV may be low, the primary vector of GIV may be a mosquito that is confined to Indonesia, the replicative ability of GIV in birds may be low, and/or the GIV transmission cycle may involve a non-migratory amplifying host [42].

### Limitations

Although valuable information was obtained from our phylogeographic study, the data should be interpreted in light of its limitations. Isolations of JEV prior to the 1970s were primarily made from humans residing in China and Japan in response to epidemic transmission of the virus. After 1970, the majority of JEV isolates were made from mosquitoes and swine throughout many prefectures of Japan (due to rare cases of JEV following introduction of an effective vaccination program) and provinces of China as part of yearly routine surveillance for JEV. Therefore, it is unlikely that the dataset is largely biased by sequences sampled from local endemic foci that would have confounded the reported genotype-climate association. Due to the small number of ORF sequence data for JEV, we utilized a dataset consisting of sequence information derived from the E gene. While the E gene of JEV was found to be a good evolutionary proxy of the ORF, we were not able to assess whether diversifying and/or directional selection in other regions of the genome may have played a role in the adaptation of the viral genotypes to their respective environments. Finally, phenotypic characterization of the five genotypes of JEV has yet to be performed and would provide further evidence to support the genotype-climate association.

### Conclusions

By applying Bayesian phylogeographic, categorical data analysis and phylogeny-trait association techniques to a large JEV sequence dataset we have demonstrated that GIII and GI-b are temperate genotypes maintained year-round in northern latitudes likely by either hibernating mosquitoes, vertical transmission in mosquitoes, poikilothermic vertebrates and/or bats. In contrast, GI-a and GII are tropical genotypes likely maintained via mosquito-avian and/or mosquito-swine transmission cycles. This suggests that the spread and establishment of GI-b throughout Asia may have been due to its ability to efficiently overwinter in temperate Asia. As highlighted by the recent emergence of West Nile virus into the western hemisphere [79] and Usutu virus into the European continent [80], the invasion of JEV into previously unoccupied regions is a real threat. Many areas of the world have JEV-competent vectors and waterbirds, and unlike West Nile and Usutu viruses, JEV also utilizes domestic swine as amplifying hosts, which can drive epidemics by producing an abundance of infected mosquitoes.

## Supporting Information

Figure S1 NJ phylogeny of the JEV sequences. The tree was rooted using the sequence of the MVE-1-51 isolate of Murray Valley encephalitis virus, which is a member of the JE serocomplex, but has been removed to allow for better visualization of branch lengths. GI-V are represented to the right of the tree. Bootstrap percentages based on 1,000 replicates are indicated at key nodes within the phylogeny. Horizontal branch lengths are proportional to the genetic distance between isolates and the scale underneath the tree indicates the number of nucleotide substitutions per site.
(TIFF)

Figure S2 ML phylogeny of the JEV sequences. The tree was rooted using the sequence of the MVE-1-51 isolate of Murray Valley encephalitis virus, which is a member of the JE serocomplex, but has been removed to allow for better visualization of branch lengths. GI-V are represented to the right of the tree. Bootstrap percentages based on 100 replicates are indicated at key nodes within the phylogeny. Horizontal branch lengths are proportional to the genetic distance between isolates and the scale beneath the tree indicates the number of nucleotide substitutions per site.
(TIFF)

Table S1 Confirmed recombinants.
(DOCX)

Table S2 Details of the JEV sequences used in this study.
(DOCX)

Table S3 Analysis of JEV phylogeographic structure.
(DOCX)

Table S4 Genotype-defining nonsynonymous substitutions within the E protein.
(DOCX)

Table S5 DEPS analysis of the JEV E protein alignment.
(DOCX)

Table S6 Amino acid sites within the E protein of JEV identified by the DEPS analyses to be under directional selection.
(DOCX)
