# Supplementary figures and images for: Phylogeography of Japanese Encephalitis Virus: Genotype Is Associated with Climate
Source: PLoS Negl Trop Dis. 2013 Aug 29;7(8):e2411. doi: 10.1371/journal.pntd.0002411 (PMC3757071; doi:10.1371/journal.pntd.0002411)

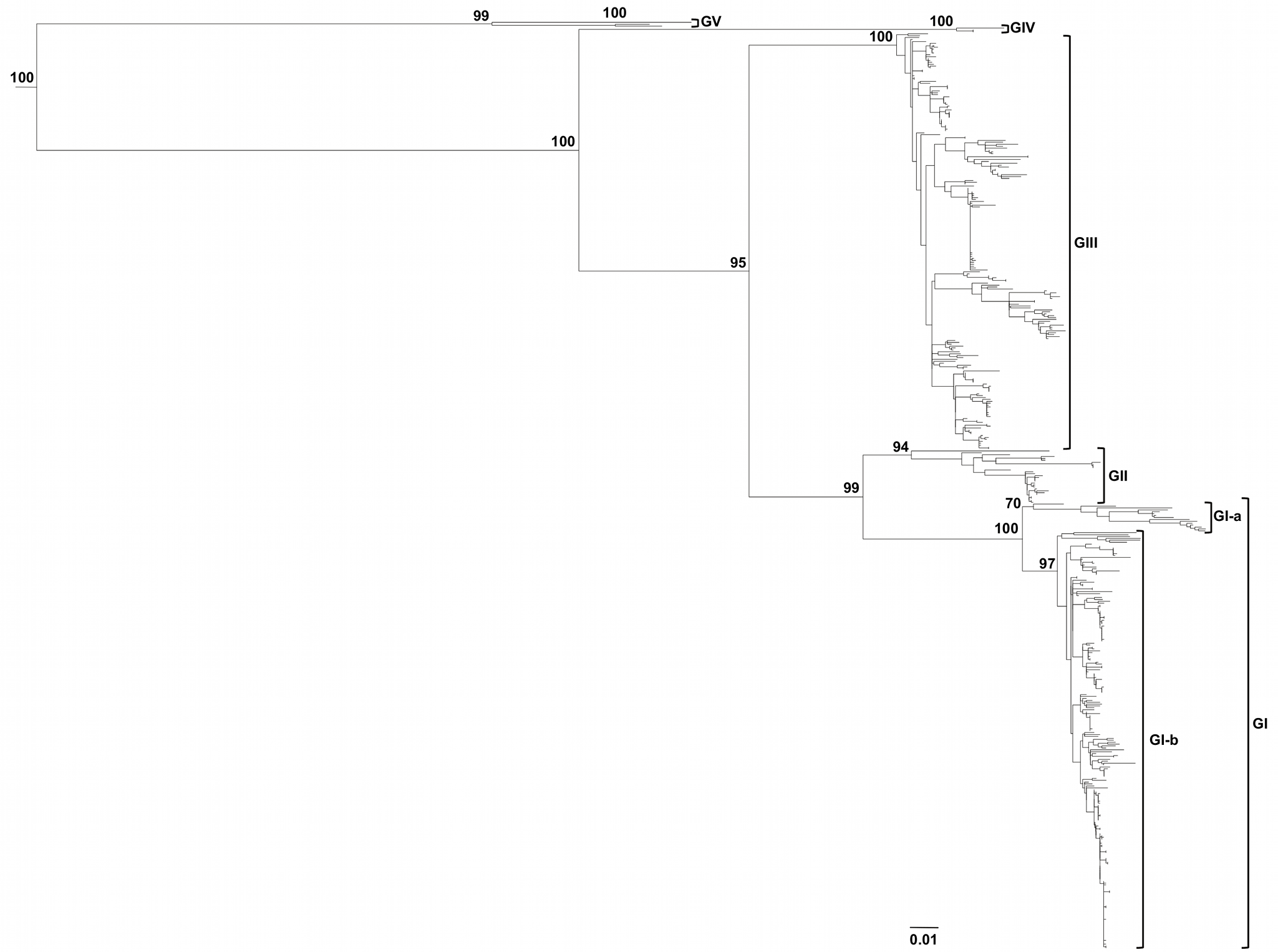

Supplement: Figure S1 — NJ phylogeny of the JEV sequences. The tree was rooted using the sequence of the MVE-1-51 isolate of Murray Valley encephalitis virus, which is a member of the JE serocomplex, but has been removed to allow for better visualization of branch lengths. GI-V are represented to the right of the tree. Bootstrap percentages based on 1,000 replicates are indicated at key nodes within the phylogeny. Horizontal branch lengths are proportional to the genetic distance between isolates and the scale underneath the tree indicates the number of nucleotide substitutions per site. (TIFF) [file pntd.0002411.s001.tiff]

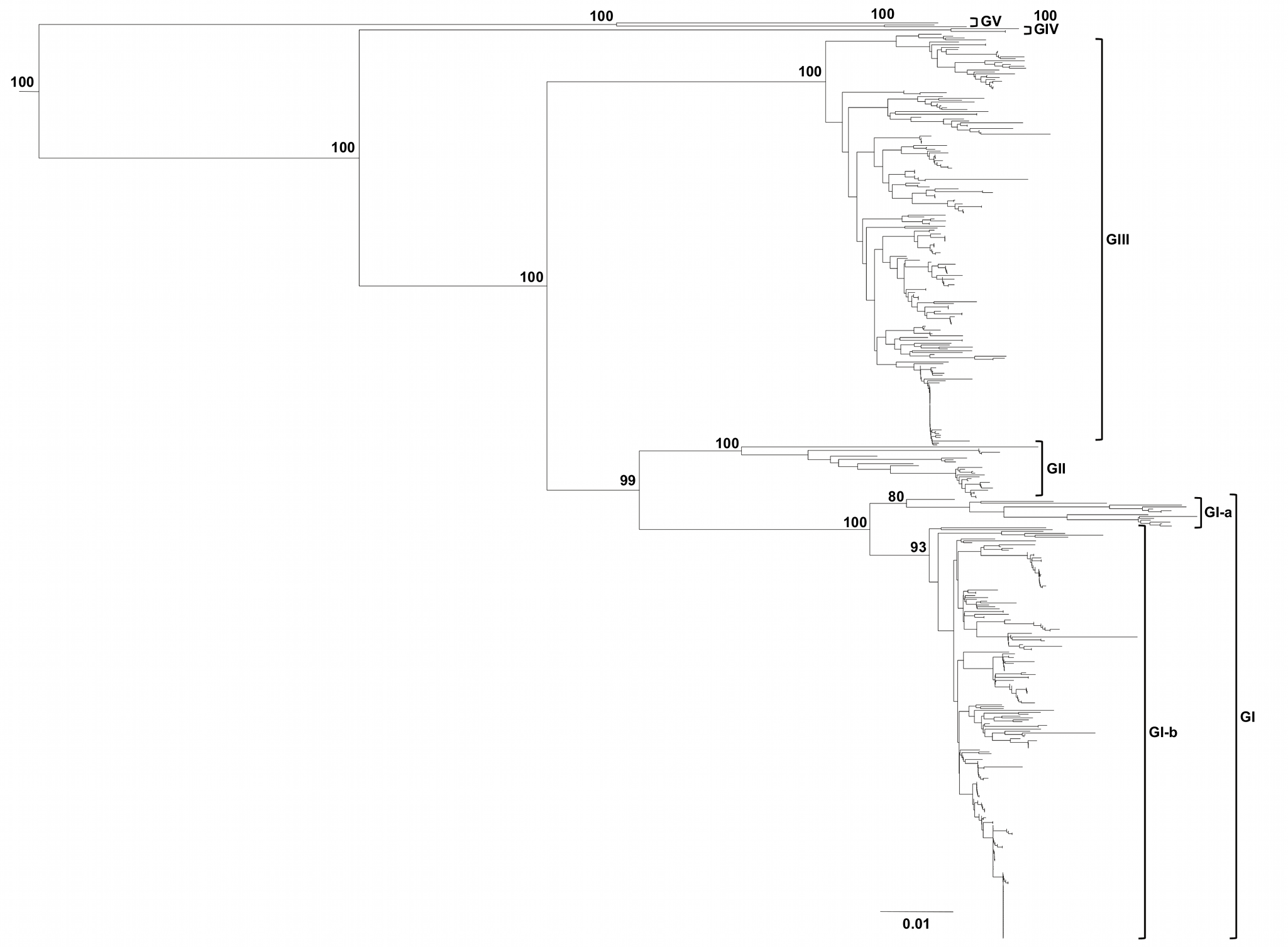

Supplement: Figure S2 — ML phylogeny of the JEV sequences. The tree was rooted using the sequence of the MVE-1-51 isolate of Murray Valley encephalitis virus, which is a member of the JE serocomplex, but has been removed to allow for better visualization of branch lengths. GI-V are represented to the right of the tree. Bootstrap percentages based on 100 replicates are indicated at key nodes within the phylogeny. Horizontal branch lengths are proportional to the genetic distance between isolates and the scale beneath the tree indicates the number of nucleotide substitutions per site. (TIFF) [file pntd.0002411.s002.tiff]
