# Supplementary material for: Phylogeography of Japanese Encephalitis Virus: Genotype Is Associated with Climate
Source: PLoS Negl Trop Dis. 2013 Aug 29;7(8):e2411. doi: 10.1371/journal.pntd.0002411 (PMC3757071; doi:10.1371/journal.pntd.0002411)
Supplement: Table S1 — Confirmed recombinants. (DOCX) [file pntd.0002411.s003.docx]

**Table S1.** Confirmed recombinants.

| Recombinant | Minor parent | Major parent | Nucleotide breakpoint positions | Algorithm^1^ |
| --- | --- | --- | --- | --- |
| K82P01 | JaNAr32-04 | JE-82 | 768-1095 | RDP |
|  |  |  |  | GENECONV |
|  |  |  |  | Bootscan |
|  |  |  |  | Maxchi |
|  |  |  |  | Chimaera |
| K91P55 | K87P39 | JaNAr32-04 | 1-298 | RDP |
|  |  |  |  | GENECONV |
|  |  |  |  | Bootscan |
|  |  |  |  | Chimaera |

^1^p < 1.0 x 10^-7^
