# Supplementary material for: Phylogeography of Japanese Encephalitis Virus: Genotype Is Associated with Climate
Source: PLoS Negl Trop Dis. 2013 Aug 29;7(8):e2411. doi: 10.1371/journal.pntd.0002411 (PMC3757071; doi:10.1371/journal.pntd.0002411)
Supplement: Table S2 — Details of the JEV sequences used in this study. (DOCX) [file pntd.0002411.s004.docx]

**Table S2.** Details of the JEV sequences used in this study.

| **Isolate** | **Country** | **Climate** | **Year** | **Host** | **Genotype** | **GenBank accession no.** |
| --- | --- | --- | --- | --- | --- | --- |
| M859/Cambodia/1967/Mosquito | Cambodia | Tropical | 1967 | Mosquito | GI-a | KF192509 |
| KE-93-83 | Thailand | Tropical | 1983 | Mosquito | GI-a | KF192510 |
| TS00 | Badu Island, Australia | Tropical | 2000 | Swine | GI-a | EF434785 |
| JE_RT_36 | Ratchaburi, Thailand | Tropical | 2003 | Mosquito | GI-a | DQ087975 |
| JE_CP_49 | Chumphon, Thailand | Tropical | 2004 | Swine | GI-a | DQ087974 |
| JE_CP_67 | Chumphon, Thailand | Tropical | 2004 | Swine | GI-a | DQ087972 |
| JE_KK_80 | Khon Khen, Thailand | Tropical | 2004 | Unknown | GI-a | DQ111784 |
| JE_KK_82 | Khon Khen, Thailand | Tropical | 2004 | Unknown | GI-a | DQ111785 |
| JE_KK_R83 | Khon Khen, Thailand | Tropical | 2004 | Unknown | GI-a | DQ111787 |
| JE_KK_R87 | Khon Khen, Thailand | Tropical | 2004 | Unknown | GI-a | DQ111788 |
| JE_KK_R88 | Khon Khen, Thailand | Tropical | 2004 | Unknown | GI-a | DQ111786 |
| JE_PK52 | Phuket, Thailand | Tropical | 2004 | Unknown | GI-a | DQ84229 |
| JE_CM_1196 | Chiang Mai, Thailand | Tropical | 2005 | Unknown | GI-a | DQ238602 |
| JE_KK_577 | Khon Khen, Thailand | Tropical | 2005 | Unknown | GI-a | DQ238601 |
| JE_KK_580 | Khon Khen, Thailand | Tropical | 2005 | Unknown | GI-a | DQ238600 |
| YN79-Bao83 | Yunnan, China | Temperate | 1979 | Mosquito | GI-b | DQ404128 |
| YN82-BN8219 | Yunnan, China | Temperate | 1982 | Mosquito | GI-b | DQ404129 |
| YN83-83199 | Yunnan, China | Temperate | 1983 | Mosquito | GI-b | DQ404131 |
| YN83-Meng83-54 | Yunnan, China | Temperate | 1983 | Midge | GI-b | DQ404130 |
| YN85-L86-99 | Yunnan, China | Temperate | 1985 | Mosquito | GI-b | DQ404132 |
| YN86-86266 | Yunnan, China | Temperate | 1986 | Unknown | GI-b | DQ404134 |
| YN86-B8639 | Yunnan, China | Temperate | 1986 | Mosquito | GI-b | DQ404133 |
| JE-91 | Korea | Temperate | 1991 | Mosquito | GI-b | GQ415355 |
| K93A07 | South Korea | Temperate | 1993 | Mosquito | GI-b | FJ938230 |
| K94P05 | Korea | Temperate | 1994 | Mosquito | GI-b | AF045551 |
| K94A07 | South Korea | Temperate | 1994 | Mosquito | GI-b | FJ938216 |
| JaTAn 1/94 | Tokyo, Japan | Temperate | 1994 | Swine | GI-b | AB237171 |
| 95-167 | Japan | Temperate | 1995 | Swine | GI-b | AY377579 |
| 95-91 | Japan | Temperate | 1995 | Swine | GI-b | AY377578 |
| 95P99 | Oita, Japan | Temperate | 1995 | Swine | GI-b | FJ943471 |
| K95A07 | South Korea | Temperate | 1995 | Mosquito | GI-b | FJ938218 |
| K96A07 | South Korea | Temperate | 1996 | Mosquito | GI-b | FJ938219 |
| 97P82 | Oita, Japan | Temperate | 1997 | Swine | GI-b | FJ943472 |
| Ishikawa | Ishikawa, Japan | Temperate | 1998 | Mosquito | GI-b | AB051292 |
| JEV/wb/Okinawa/1/1998 | Okinawa, Japan | Temperate | 1998 | Swine | GI-b | AB306941 |
| 99P103 | Oita, Japan | Temperate | 1999 | Swine | GI-b | FJ943473 |
| 99P104 | Oita, Japan | Temperate | 1999 | Swine | GI-b | FJ943474 |
| KV1899 | South Korea | Temperate | 1999 | Swine | GI-b | AF474075 |
| SH-101 | Shanghai, China | Temperate | 2001 | Mosquito | GI-b | AY555761 |
| SH-53 | Shanghai, China | Temperate | 2001 | Mosquito | GI-b | AY555757 |
| SH-80 | Shanghai, China | Temperate | 2001 | Mosquito | GI-b | AY243841 |
| SH-81 | Shanghai, China | Temperate | 2001 | Mosquito | GI-b | AY555758 |
| SH-83 | Shanghai, China | Temperate | 2001 | Mosquito | GI-b | AY555759 |
| SH-90 | Shanghai, China | Temperate | 2001 | Mosquito | GI-b | AY243835 |
| SH-96 | Shanghai, China | Temperate | 2001 | Mosquito | GI-b | AY555760 |
| K01-GN | South Korea | Temperate | 2001 | Mosquito | GI-b | FJ938220 |
| K01-JB | South Korea | Temperate | 2001 | Mosquito | GI-b | FJ938221 |
| K01-JN | South Korea | Temperate | 2001 | Mosquito | GI-b | FJ938222 |
| VN88 | Vietnam | Tropical | 2001 | Swine | GI-b | AY376464 |
| JEV/sw/Chiba/88/2002 | Chiba, Japan | Temperate | 2002 | Swine | GI-b | AB112705 |
| JEV/sw/Hiroshima/25/2002 | Hiroshima, Japan | Temperate | 2002 | Swine | GI-b | AB231465 |
| JaNAr0102 | Japan | Temperate | 2002 | Mosquito | GI-b | AY377577 |
| JEV/sw/Kagawa/24/2002 | Kagawa, Japan | Temperate | 2002 | Swine | GI-b | AB112706 |
| JEV/sw/Kagawa/27/2002 | Kagawa, Japan | Temperate | 2002 | Swine | GI-b | AB112707 |
| LN02-102 | Liaoning, China | Temperate | 2002 | Mosquito | GI-b | DQ404085 |
| LN02-104 | Liaoning, China | Temperate | 2002 | Mosquito | GI-b | DQ404086 |
| JEV/sw/Mie/41/2002 | Mie, Japan | Temperate | 2002 | Swine | GI-b | AB112709 |
| JEV/sw/Shizuoka/33/2002 | Shizuoka, Japan | Temperate | 2002 | Swine | GI-b | AB112703 |
| JEV/sw/Shizuoka/39/2002 | Shizuoka, Japan | Temperate | 2002 | Swine | GI-b | AB112704 |
| VN105 | Vietnam | Tropical | 2002 | Mosquito | GI-b | AY376468 |
| VN22 | Vietnam | Tropical | 2002 | Swine | GI-b | AY376465 |
| VN34 | Vietnam | Tropical | 2002 | Mosquito | GI-b | AY376466 |
| VN78 | Vietnam | Tropical | 2002 | Mosquito | GI-b | AY376467 |
| 03P113 | Oita, Japan | Temperate | 2003 | Swine | GI-b | FJ943475 |
| 03P120 | Oita, Japan | Temperate | 2003 | Swine | GI-b | FJ943476 |
| 03P126 | Oita, Japan | Temperate | 2003 | Swine | GI-b | FJ943477 |
| 03P145 | Oita, Japan | Temperate | 2003 | Swine | GI-b | FJ943478 |
| 03P189 | Oita, Japan | Temperate | 2003 | Swine | GI-b | FJ943479 |
| JEV/sw/Okinawa/285/2003 | Okinawa, Japan | Temperate | 2003 | Swine | GI-b | AB238693 |
| SH03-103 | Shanghai, China | Temperate | 2003 | Mosquito | GI-b | DQ404096 |
| SH03-105 | Shanghai, China | Temperate | 2003 | Mosquito | GI-b | DQ404097 |
| SH03-109 | Shanghai, China | Temperate | 2003 | Mosquito | GI-b | DQ404098 |
| SH03-115 | Shanghai, China | Temperate | 2003 | Mosquito | GI-b | DQ404099 |
| SH03-124 | Shanghai, China | Temperate | 2003 | Mosquito | GI-b | DQ404100 |
| SH03-127 | Shanghai, China | Temperate | 2003 | Mosquito | GI-b | DQ404101 |
| SH03-128 | Shanghai, China | Temperate | 2003 | Mosquito | GI-b | DQ404102 |
| SH03-129 | Shanghai, China | Temperate | 2003 | Mosquito | GI-b | DQ404103 |
| SH03-130 | Shanghai, China | Temperate | 2003 | Mosquito | GI-b | DQ404104 |
| JEV/eq/Tottori/2003 | Tottori, Japan | Temperate | 2003 | Equid | GI-b | AB213007 |
| HN04-11 | Henan, China | Temperate | 2004 | Mosquito | GI-b | DQ404087 |
| HN04-21 | Henan, China | Temperate | 2004 | Mosquito | GI-b | DQ404088 |
| HN04-40 | Henan, China | Temperate | 2004 | Mosquito | GI-b | DQ404089 |
| JaNAr07-04 | Isahaya, Nagasaki Prefecture, Japan | Temperate | 2004 | Mosquito | GI-b | FJ185144 |
| JaNAr10-04 | Isahaya, Nagasaki Prefecture, Japan | Temperate | 2004 | Mosquito | GI-b | FJ185145 |
| JaNAr13-04 | Isahaya, Nagasaki Prefecture, Japan | Temperate | 2004 | Mosquito | GI-b | FJ185146 |
| JaNAr31-04 | Isahaya, Nagasaki Prefecture, Japan | Temperate | 2004 | Mosquito | GI-b | FJ185150 |
| JaNAr32-04 | Isahaya, Nagasaki Prefecture, Japan | Temperate | 2004 | Mosquito | GI-b | FJ185151 |
| JaNAr38-04 | Isahaya, Nagasaki Prefecture, Japan | Temperate | 2004 | Mosquito | GI-b | FJ185152 |
| JEV/sw/Kagawa/35/2004 | Kagawa, Japan | Temperate | 2004 | Swine | GI-b | AB231464 |
| JEV/sw/Mie/34/2004 | Mie, Japan | Temperate | 2004 | Swine | GI-b | AB231462 |
| JEV/sw/Mie/40/2004 | Mie, Japan | Temperate | 2004 | Swine | GI-b | AB231463 |
| SC04-12 | Sichuan, China | Temperate | 2004 | Mosquito | GI-b | DQ404090 |
| SC04-15 | Sichuan, China | Temperate | 2004 | Mosquito | GI-b | DQ404091 |
| SC04-16 | Sichuan, China | Temperate | 2004 | Mosquito | GI-b | DQ404092 |
| SC04-17 | Sichuan, China | Temperate | 2004 | Mosquito | GI-b | DQ404093 |
| SC04-25 | Sichuan, China | Temperate | 2004 | Mosquito | GI-b | DQ404094 |
| SC04-27 | Sichuan, China | Temperate | 2004 | Mosquito | GI-b | DQ404095 |
| GX0519 | Guangxi, China | Temperate | 2005 | Mosquito | GI-b | FJ161967 |
| GX0523 | Guangxi, China | Temperate | 2005 | Mosquito | GI-b | FJ161968 |
| GX0558 | Guangxi, China | Temperate | 2005 | Mosquito | GI-b | FJ161969 |
| JE_KK_1116 | Khon Khen, Thailand | Tropical | 2005 | Unknown | GI-b | DQ343290 |
| 05P75 | Oita, Japan | Temperate | 2005 | Swine | GI-b | FJ943480 |
| SH05-24 | Shanghai, China | Temperate | 2005 | Mosquito | GI-b | DQ404108 |
| K05-GS | South Korea | Temperate | 2005 | Mosquito | GI-b | FJ938223 |
| Mo/Toyama/1089c/2005 | Toyama, Japan | Temperate | 2005 | Mosquito | GI-b | AB538603 |
| Mo/Toyama/1089v/2005 | Toyama, Japan | Temperate | 2005 | Mosquito | GI-b | AB538604 |
| Mo/Toyama/1148c/2005 | Toyama, Japan | Temperate | 2005 | Mosquito | GI-b | AB538605 |
| Mo/Toyama/1148v/2005 | Toyama, Japan | Temperate | 2005 | Mosquito | GI-b | AB538606 |
| Mo/Toyama/1149c/2005 | Toyama, Japan | Temperate | 2005 | Mosquito | GI-b | AB538607 |
| Mo/Toyama/1155c/2005 | Toyama, Japan | Temperate | 2005 | Mosquito | GI-b | AB538608 |
| Mo/Toyama/1155v/2005 | Toyama, Japan | Temperate | 2005 | Mosquito | GI-b | AB538609 |
| Mo/Toyama/1157c/2005 | Toyama, Japan | Temperate | 2005 | Mosquito | GI-b | AB538610 |
| Mo/Toyama/1158c/2005 | Toyama, Japan | Temperate | 2005 | Mosquito | GI-b | AB538611 |
| Mo/Toyama/1158v/2005 | Toyama, Japan | Temperate | 2005 | Mosquito | GI-b | AB538612 |
| Mo/Toyama/1160c/2005 | Toyama, Japan | Temperate | 2005 | Mosquito | GI-b | AB538613 |
| Mo/Toyama/1161c/2005 | Toyama, Japan | Temperate | 2005 | Mosquito | GI-b | AB538614 |
| Mo/Toyama/1161v/2005 | Toyama, Japan | Temperate | 2005 | Mosquito | GI-b | AB538615 |
| Mo/Toyama/1222c/2005 | Toyama, Japan | Temperate | 2005 | Mosquito | GI-b | AB538616 |
| Mo/Toyama/1222v/2005 | Toyama, Japan | Temperate | 2005 | Mosquito | GI-b | AB538617 |
| Mo/Toyama/1256c/2005 | Toyama, Japan | Temperate | 2005 | Mosquito | GI-b | AB538618 |
| Mo/Toyama/1256v/2005 | Toyama, Japan | Temperate | 2005 | Mosquito | GI-b | AB538619 |
| Sw/Toyama/05197v/2005 | Toyama, Japan | Temperate | 2005 | Swine | GI-b | AB538823 |
| Sw/Toyama/05231v/2005 | Toyama, Japan | Temperate | 2005 | Swine | GI-b | AB538824 |
| Mo/Toyama/1018c/2005 | Toyama, Japan | Temperate | 2005 | Mosquito | GI-b | AB538601 |
| Mo/Toyama/1018v/2005 | Toyama, Japan | Temperate | 2005 | Mosquito | GI-b | AB538602 |
| CT-MO-P7 | Vietnam | Tropical | 2005 | Swine | GI-b | HQ009266 |
| LA_H06-05 | Vietnam | Tropical | 2005 | Mosquito | GI-b | FJ185153 |
| LA_H07-05 | Vietnam | Tropical | 2005 | Mosquito | GI-b | FJ185154 |
| LA-H-5330 | Vietnam | Tropical | 2005 | Swine | GI-b | HQ009265 |
| LAH_2079-05 | Vietnam | Tropical | 2005 | Mosquito | GI-b | FJ185155 |
| 06P152 | Oita, Japan | Temperate | 2006 | Swine | GI-b | FJ943481 |
| 06P183 | Oita, Japan | Temperate | 2006 | Swine | GI-b | FJ943483 |
| 06P212 | Oita, Japan | Temperate | 2006 | Swine | GI-b | FJ943484 |
| HEN0701 | Henan, China | Temperate | 2007 | Swine | GI-b | FJ156730 |
| JaNAr06-07 | Isahaya, Nagasaki Prefecture, Japan | Temperate | 2007 | Mosquito | GI-b | FJ185143 |
| JaNAr14-07 | Isahaya, Nagasaki Prefecture, Japan | Temperate | 2007 | Mosquito | GI-b | FJ185147 |
| JaNAr15-07 | Isahaya, Nagasaki Prefecture, Japan | Temperate | 2007 | Mosquito | GI-b | FJ185148 |
| JaNAr17-07 | Isahaya, Nagasaki Prefecture, Japan | Temperate | 2007 | Mosquito | GI-b | FJ185149 |
| 07P127 | Oita, Japan | Temperate | 2007 | Swine | GI-b | FJ943487 |
| 07P83 | Oita, Japan | Temperate | 2007 | Swine | GI-b | FJ943485 |
| 07P90 | Oita, Japan | Temperate | 2007 | Swine | GI-b | FJ943486 |
| Mo/Toyama/2347c/2007 | Toyama, Japan | Temperate | 2007 | Mosquito | GI-b | AB538658 |
| Mo/Toyama/2441c/2007 | Toyama, Japan | Temperate | 2007 | Mosquito | GI-b | AB538659 |
| Mo/Toyama/2462c/2007 | Toyama, Japan | Temperate | 2007 | Mosquito | GI-b | AB538660 |
| Mo/Toyama/2506c/2007 | Toyama, Japan | Temperate | 2007 | Mosquito | GI-b | AB538661 |
| Mo/Toyama/2507c/2007 | Toyama, Japan | Temperate | 2007 | Mosquito | GI-b | AB538662 |
| Mo/Toyama/2513c/2007 | Toyama, Japan | Temperate | 2007 | Mosquito | GI-b | AB538663 |
| Mo/Toyama/2513v/2007 | Toyama, Japan | Temperate | 2007 | Mosquito | GI-b | AB538664 |
| Mo/Toyama/2554c/2007 | Toyama, Japan | Temperate | 2007 | Mosquito | GI-b | AB538665 |
| Mo/Toyama/2554v/2007 | Toyama, Japan | Temperate | 2007 | Mosquito | GI-b | AB538666 |
| Mo/Toyama/2556c/2007 | Toyama, Japan | Temperate | 2007 | Mosquito | GI-b | AB538667 |
| Mo/Toyama/2556v/2007 | Toyama, Japan | Temperate | 2007 | Mosquito | GI-b | AB538668 |
| Mo/Toyama/2567c/2007 | Toyama, Japan | Temperate | 2007 | Mosquito | GI-b | AB538669 |
| Mo/Toyama/2569c/2007 | Toyama, Japan | Temperate | 2007 | Mosquito | GI-b | AB538670 |
| Mo/Toyama/2569v/2007 | Toyama, Japan | Temperate | 2007 | Mosquito | GI-b | AB538671 |
| Sw/Toyama/07232c/2007 | Toyama, Japan | Temperate | 2007 | Swine | GI-b | AB538825 |
| Sw/Toyama/07234c/2007 | Toyama, Japan | Temperate | 2007 | Swine | GI-b | AB538826 |
| Sw/Toyama/07240c/2007 | Toyama, Japan | Temperate | 2007 | Swine | GI-b | AB538827 |
| Sw/Toyama/07292c/2007 | Toyama, Japan | Temperate | 2007 | Swine | GI-b | AB538828 |
| Sw/Toyama/07292v/2007 | Toyama, Japan | Temperate | 2007 | Swine | GI-b | AB538829 |
| Sw/Toyama/07296c/2007 | Toyama, Japan | Temperate | 2007 | Swine | GI-b | AB538830 |
| Sw/Toyama/07326c/2007 | Toyama, Japan | Temperate | 2007 | Swine | GI-b | AB538831 |
| 07VN310 | Vietnam | Tropical | 2007 | Mosquito | GI-b | HM228922 |
| 07VN311 | Vietnam | Tropical | 2007 | Mosquito | GI-b | HM228923 |
| XJ69 | Zhejiang, China | Temperate | 2007 | Mosquito | GI-b | EU258742 |
| XJP613 | Zhejiang, China | Temperate | 2007 | Mosquito | GI-b | EU258741 |
| Japanese wild boar | Hyogo, Nishinomiya Prefecture, Japan | Temperate | 2008 | Swine | GI-b | AB481224 |
| 08P37 | Oita, Japan | Temperate | 2008 | Swine | GI-b | FJ943488 |
| 08P38 | Oita, Japan | Temperate | 2008 | Swine | GI-b | FJ943489 |
| 08P42 | Oita, Japan | Temperate | 2008 | Swine | GI-b | FJ943490 |
| 08P48 | Oita, Japan | Temperate | 2008 | Swine | GI-b | FJ943491 |
| 08P49 | Oita, Japan | Temperate | 2008 | Swine | GI-b | FJ943492 |
| 08P54 | Oita, Japan | Temperate | 2008 | Swine | GI-b | FJ943493 |
| 08P62 | Oita, Japan | Temperate | 2008 | Swine | GI-b | FJ943494 |
| JEV/sw/Okinawa/154/2008 | Okinawa, Japan | Temperate | 2008 | Swine | GI-b | AB471666 |
| JEV/sw/Okinawa/254/2008 | Okinawa, Japan | Temperate | 2008 | Swine | GI-b | AB471667 |
| JEV/sw/Okinawa/372/2008 | Okinawa, Japan | Temperate | 2008 | Swine | GI-b | AB471668 |
| JEV/sw/Okinawa/377/2008 | Okinawa, Japan | Temperate | 2008 | Swine | GI-b | AB471669 |
| JEV/sw/Okinawa/402/2008 | Okinawa, Japan | Temperate | 2008 | Swine | GI-b | AB471670 |
| TPC0806c | Taipei County, Taiwan | Temperate | 2008 | Mosquito | GI-b | GQ260635 |
| Mo/Toyama/2757c/2008 | Toyama, Japan | Temperate | 2008 | Mosquito | GI-b | AB538700 |
| Mo/Toyama/2759c/2008 | Toyama, Japan | Temperate | 2008 | Mosquito | GI-b | AB538701 |
| Mo/Toyama/2794c/2008 | Toyama, Japan | Temperate | 2008 | Mosquito | GI-b | AB538702 |
| Mo/Toyama/2794v/2008 | Toyama, Japan | Temperate | 2008 | Mosquito | GI-b | AB538703 |
| Mo/Toyama/2795c/2008 | Toyama, Japan | Temperate | 2008 | Mosquito | GI-b | AB538704 |
| Mo/Toyama/2795v/2008 | Toyama, Japan | Temperate | 2008 | Mosquito | GI-b | AB538705 |
| Mo/Toyama/2805c/2008 | Toyama, Japan | Temperate | 2008 | Mosquito | GI-b | AB538706 |
| Mo/Toyama/2805v/2008 | Toyama, Japan | Temperate | 2008 | Mosquito | GI-b | AB538707 |
| Mo/Toyama/2808c/2008 | Toyama, Japan | Temperate | 2008 | Mosquito | GI-b | AB538708 |
| Mo/Toyama/2821c/2008 | Toyama, Japan | Temperate | 2008 | Mosquito | GI-b | AB538709 |
| Mo/Toyama/2842c/2008 | Toyama, Japan | Temperate | 2008 | Mosquito | GI-b | AB538710 |
| Mo/Toyama/2842v/2008 | Toyama, Japan | Temperate | 2008 | Mosquito | GI-b | AB538711 |
| Mo/Toyama/2853c/2008 | Toyama, Japan | Temperate | 2008 | Mosquito | GI-b | AB538712 |
| Mo/Toyama/2853v/2008 | Toyama, Japan | Temperate | 2008 | Mosquito | GI-b | AB538713 |
| Mo/Toyama/2886c/2008 | Toyama, Japan | Temperate | 2008 | Mosquito | GI-b | AB538714 |
| Mo/Toyama/2886v/2008 | Toyama, Japan | Temperate | 2008 | Mosquito | GI-b | AB538715 |
| Mo/Toyama/2888c/2008 | Toyama, Japan | Temperate | 2008 | Mosquito | GI-b | AB538716 |
| Mo/Toyama/2895c/2008 | Toyama, Japan | Temperate | 2008 | Mosquito | GI-b | AB538717 |
| Mo/Toyama/2905c/2008 | Toyama, Japan | Temperate | 2008 | Mosquito | GI-b | AB538718 |
| Mo/Toyama/2906c/2008 | Toyama, Japan | Temperate | 2008 | Mosquito | GI-b | AB538719 |
| Mo/Toyama/2909c/2008 | Toyama, Japan | Temperate | 2008 | Mosquito | GI-b | AB538720 |
| Mo/Toyama/2910c/2008 | Toyama, Japan | Temperate | 2008 | Mosquito | GI-b | AB538721 |
| Mo/Toyama/2915c/2008 | Toyama, Japan | Temperate | 2008 | Mosquito | GI-b | AB538722 |
| Mo/Toyama/2917c/2008 | Toyama, Japan | Temperate | 2008 | Mosquito | GI-b | AB538723 |
| Mo/Toyama/2918c/2008 | Toyama, Japan | Temperate | 2008 | Mosquito | GI-b | AB538724 |
| Mo/Toyama/2929c/2008 | Toyama, Japan | Temperate | 2008 | Mosquito | GI-b | AB538725 |
| Mo/Toyama/2929v/2008 | Toyama, Japan | Temperate | 2008 | Mosquito | GI-b | AB538726 |
| Mo/Toyama/2967c/2008 | Toyama, Japan | Temperate | 2008 | Mosquito | GI-b | AB538727 |
| Mo/Toyama/2967v/2008 | Toyama, Japan | Temperate | 2008 | Mosquito | GI-b | AB538728 |
| Mo/Toyama/2976c/2008 | Toyama, Japan | Temperate | 2008 | Mosquito | GI-b | AB538729 |
| Mo/Toyama/2976v/2008 | Toyama, Japan | Temperate | 2008 | Mosquito | GI-b | AB538730 |
| Mo/Toyama/2977c/2008 | Toyama, Japan | Temperate | 2008 | Mosquito | GI-b | AB538731 |
| Mo/Toyama/2977v/2008 | Toyama, Japan | Temperate | 2008 | Mosquito | GI-b | AB538732 |
| Mo/Toyama/2984c/2008 | Toyama, Japan | Temperate | 2008 | Mosquito | GI-b | AB538733 |
| Mo/Toyama/2984v/2008 | Toyama, Japan | Temperate | 2008 | Mosquito | GI-b | AB538734 |
| Mo/Toyama/2985c/2008 | Toyama, Japan | Temperate | 2008 | Mosquito | GI-b | AB538735 |
| Mo/Toyama/2985v/2008 | Toyama, Japan | Temperate | 2008 | Mosquito | GI-b | AB538736 |
| Mo/Toyama/2986c/2008 | Toyama, Japan | Temperate | 2008 | Mosquito | GI-b | AB538737 |
| Mo/Toyama/2986v/2008 | Toyama, Japan | Temperate | 2008 | Mosquito | GI-b | AB538738 |
| Mo/Toyama/2987c/2008 | Toyama, Japan | Temperate | 2008 | Mosquito | GI-b | AB538739 |
| Mo/Toyama/2987v/2008 | Toyama, Japan | Temperate | 2008 | Mosquito | GI-b | AB538740 |
| Sw/Toyama/08253c/2008 | Toyama, Japan | Temperate | 2008 | Swine | GI-b | AB538832 |
| YL0806f | Yilan County, Taiwan | Temperate | 2008 | Mosquito | GI-b | GQ260633 |
| FQ24M-08 | Yunnan, China | Temperate | 2008 | Mosquito | GI-b | HM204531 |
| XP174M-08 | Yunnan, China | Temperate | 2008 | Mosquito | GI-b | HM204527 |
| 09P123 | Oita, Japan | Temperate | 2009 | Swine | GI-b | GU108334 |
| 09P141 | Oita, Japan | Temperate | 2009 | Swine | GI-b | GU108335 |
| LY5P-09 | Shanxi, China | Temperate | 2009 | Human | GI-b | HM204530 |
| JEV-CZ1 | Sichuan, China | Temperate | 2009 | Mosquito | GI-b | HM234673 |
| Mo/Toyama/3133c/2009 | Toyama, Japan | Temperate | 2009 | Mosquito | GI-b | AB543738 |
| Mo/Toyama/3140c/2009 | Toyama, Japan | Temperate | 2009 | Mosquito | GI-b | AB543739 |
| Mo/Toyama/3141c/2009 | Toyama, Japan | Temperate | 2009 | Mosquito | GI-b | AB543740 |
| LX10P-09 | Yunnan, China | Temperate | 2009 | Human | GI-b | HM204528 |
| LX29P-09 | Yunnan, China | Temperate | 2009 | Human | GI-b | HM204529 |
| Bennett | Korea | Temperate | 1951 | Human | GII | FJ872376 |
| WTP-70-22 | Malaysia | Tropical | 1970 | Mosquito | GII | HQ223286 |
| DjAr703 | West Java, Indonesia | Tropical | 1974 | Mosquito | GII |  |
| JKT654 | Indonesia | Tropical | 1978 | Mosquito | GII | HQ223287 |
| JKT1724 | Indonesia | Tropical | 1979 | Mosquito | GII | JQ429304 |
| JKT1749 | Indonesia | Tropical | 1979 | Mosquito | GII | JQ429305 |
| JKT220507 | Jakarta, Java, Indonesia | Tropical | 1979 | Mosquito | GII | JQ429291 |
| JKT1110 | Kapuk, Java, Indonesia | Tropical | 1979 | Mosquito | GII | JQ429288 |
| JKT1729 | Kapuk, Java, Indonesia | Tropical | 1979 | Mosquito | GII | JQ429289 |
| JKT1754 | Kapuk, Java, Indonesia | Tropical | 1979 | Mosquito | GII | JQ429290 |
| JKT2212 | Kapuk, Java, Indonesia | Tropical | 1979 | Mosquito | GII | JQ429292 |
| JKT2303 | Kapuk, Java, Indonesia | Tropical | 1979 | Mosquito | GII | JQ429295 |
| JKT2329 | Kapuk, Java, Indonesia | Tropical | 1979 | Mosquito | GII | JQ429298 |
| JKT2352 | Kapuk, Java, Indonesia | Tropical | 1979 | Mosquito | GII | JQ429299 |
| JKT2362 | Kapuk, Java, Indonesia | Tropical | 1979 | Mosquito | GII | JQ429296 |
| JKT2380 | Kapuk, Java, Indonesia | Tropical | 1979 | Mosquito | GII | JQ429297 |
| JKT4312 | Kapuk, Java, Indonesia | Tropical | 1979 | Mosquito | GII | JQ429300 |
| JKT4331 | Kapuk, Java, Indonesia | Tropical | 1979 | Mosquito | GII | JQ429301 |
| JKT4332 | Kapuk, Java, Indonesia | Tropical | 1979 | Mosquito | GII | JQ429302 |
| JKT811 | Kapuk, Java, Indonesia | Tropical | 1979 | Mosquito | GII | JQ429303 |
| JKT2254 | Lombok, Indonesia | Tropical | 1979 | Mosquito | GII | JQ429293 |
| JKT2267 | Lombok, Indonesia | Tropical | 1979 | Mosquito | GII | JQ429294 |
| JKT5441 | Indonesia | Tropical | 1980 | Mosquito | GII | JQ429306 |
| FU | Australia | Tropical | 1995 | Human | GII | L43565 |
| M15 | Australia | Tropical | 1995 | Unknown | GII | L47439 |
| M40 | Australia | Tropical | 1995 | Unknown | GII | L47350 |
| NO | Australia | Tropical | 1995 | Unknown | GII | L43566 |
| CNS138-11 | Malaysia | Tropical | 1999 | Human | GII | AY184213 |
| Nakayama | Nakayama, Japan | Temperate | 1935 | Human | GIII | EF571853 |
| Matsunaga | Japan | Temperate | 1939 | Human | GIII | FJ872381 |
| Roum | Korea | Temperate | 1946 | Human | GIII | FJ872377 |
| Taira | Japan | Temperate | 1948 | Human | GIII | FJ872384 |
| Beijing-1 | Beijing, China | Temperate | 1949 | Mosquito | GIII | L48961 |
| Equine | Japan | Temperate | 1949 | Equid | GIII | FJ872378 |
| K-29 | Korea | Temperate | 1949 | Human | GIII | GQ415356 |
| V9-3901 | Japan | Temperate | 1950 | Human | GIII | FJ872382 |
| V9-3902 | Japan | Temperate | 1950 | Human | GIII | FJ872383 |
| V9-4399 | Japan | Temperate | 1950 | Human | GIII | FJ872380 |
| Korea Jap B | Korea | Temperate | 1950 | Human | GIII | FJ872379 |
| JaOArK151 | Oita Prefecture, Japan | Temperate | 1951 | Unknown | GIII | AB028255 |
| SA14 | China | Temperate | 1954 | Mosquito | GIII | U14163 |
| CBH | Fujian, China | Temperate | 1954 | Human | GIII | DQ404116 |
| CZX | Fujian, China | Temperate | 1954 | Human | GIII | AY243828 |
| G35 | Fujian, China | Temperate | 1954 | Mosquito | GIII | AY243831 |
| CTS | Fujian, China | Temperate | 1955 | Human | GIII | AY243830 |
| LFM | Fujian, China | Temperate | 1955 | Human | GIII | AY243833 |
| YLG | Fujian, China | Temperate | 1955 | Human | GIII | AY243837 |
| ZMT | Fujian, China | Temperate | 1955 | Human | GIII | AY243840 |
| ZSZ | Fujian, China | Temperate | 1955 | Human | GIII | AY243839 |
| LYZ | Fujian, China | Temperate | 1957 | Human | GIII | AY243834 |
| CH-13 | Sichuan, China | Temperate | 1957 | Human | GIII | AY243829 |
| HVI | Taiwan | Unknown | 1958 | Mosquito | GIII | AF098735 |
| JaTH160 | Tokyo, Japan | Temperate | 1960 | Human | GIII | AB269326 |
| JaGAr01 | Japan | Temperate | 1965 | Human | GIII | U44964 |
| Ling | Ling, Taiwan | Temperate | 1965 | Human | GIII | L78128 |
| TC | Taiwan | Unknown | 1965 | Mosquito | GIII | AF098736 |
| TL | Taiwan | Unknown | 1965 | Mosquito | GIII | AF098737 |
| JaOH0566 | Japan | Temperate | 1966 | Human | GIII | AY029207 |
| Kamiyama 1 | Kamiyama, Japan | Temperate | 1966 | Human | GIII | S47265 |
| JaOAr404 | Oita Prefecture, Japan | Temperate | 1968 | Unknown | GIII | AB028250 |
| Oita100-69 | Oita Prefecture, Japan | Temperate | 1969 | Unknown | GIII | AB028269 |
| 691004 | Sri Lanka | Tropical | 1969 | Human | GIII | Z34097 |
| MIe731-70 | Mie, Japan | Temperate | 1970 | Unknown | GIII | AB028271 |
| JaNAr516-70 | Nagasaki Prefecture, Japan | Temperate | 1970 | Unknown | GIII | AB028270 |
| JaOAr363 | Oita Prefecture, Japan | Temperate | 1970 | Unknown | GIII | AB028252 |
| Tla | Heilongjiang, China | Temperate | 1971 | Human | GIII | AY243826 |
| JaOAr72 | Okinawa, Japan | Temperate | 1972 | Mosquito | GIII | AB569990 |
| 733913 | Bankura, West Bengal, India | Tropical | 1973 | Human | GIII | EU372660 |
| JaOAr73050 | Okinawa, Japan | Temperate | 1973 | Swine | GIII | AB569987 |
| JaOAr73055 | Okinawa, Japan | Temperate | 1973 | Swine | GIII | AB569988 |
| JaOAr73062 | Okinawa, Japan | Temperate | 1973 | Swine | GIII | AB569989 |
| JaOS73620 | Okinawa, Japan | Temperate | 1973 | Swine | GIII | AB569978 |
| JaOS73832 | Okinawa, Japan | Temperate | 1973 | Swine | GIII | AB569979 |
| JaOAr74010 | Okinawa, Japan | Temperate | 1974 | Mosquito | GIII | AB569994 |
| JaOAr74011 | Okinawa, Japan | Temperate | 1974 | Mosquito | GIII | AB569995 |
| JaOS74728 | Okinawa, Japan | Temperate | 1974 | Swine | GIII | AB569992 |
| JaOS74729 | Okinawa, Japan | Temperate | 1974 | Swine | GIII | AB569993 |
| JaOS74801 | Okinawa, Japan | Temperate | 1974 | Swine | GIII | AB569996 |
| JaOS75571 | Okinawa, Japan | Temperate | 1975 | Swine | GIII | AB569980 |
| JaOS75642 | Okinawa, Japan | Temperate | 1975 | Swine | GIII | AB569981 |
| JaOS75672 | Okinawa, Japan | Temperate | 1975 | Swine | GIII | AB569982 |
| JaOS75722 | Okinawa, Japan | Temperate | 1975 | Swine | GIII | AB569983 |
| JaOS75770 | Okinawa, Japan | Temperate | 1975 | Swine | GIII | AB569984 |
| JaOS75833 | Okinawa, Japan | Temperate | 1975 | Swine | GIII | AB569985 |
| JaOS75918 | Okinawa, Japan | Temperate | 1975 | Swine | GIII | AB569986 |
| JaOAr76075 | Okinawa, Japan | Temperate | 1976 | Mosquito | GIII | AB569991 |
| GP78 | Gorakhpur, India | Temperate | 1978 | Mosquito | GIII | AF075723 |
| Liyujie | Yunnan, China | Temperate | 1979 | Human | GIII | FJ185039 |
| 80P136 | Japan | Temperate | 1980 | Swine | GIII | FJ943462 |
| 80P205 | Japan | Temperate | 1980 | Swine | GIII | FJ943463 |
| 81P241 | Japan | Temperate | 1981 | Swine | GIII | FJ943464 |
| 81P244 | Japan | Temperate | 1981 | Swine | GIII | FJ943465 |
| JE-82 | Korea | Temperate | 1982 | Mosquito | GIII | GQ415347 |
| JaOArS982 | Osaka, Japan | Temperate | 1982 | Mosquito | GIII | NC_001437 |
| BN19 | Yunnan, China | Temperate | 1982 | Mosquito | GIII | FJ185038 |
| JE-83 | Korea | Temperate | 1983 | Mosquito | GIII | GQ415348 |
| cc27 | Pingtun County, Taiwan | Tropical | 1983 | Mosquito | GIII | U44957 |
| K83P34 | South Korea | Temperate | 1983 | Mosquito | GIII | FJ938231 |
| K83P44 | South Korea | Temperate | 1983 | Mosquito | GIII | FJ938232 |
| JE-84 | Korea | Temperate | 1984 | Mosquito | GIII | GQ415349 |
| CC94 | Pingtun County, Taiwan | Tropical | 1984 | Mosquito | GIII | U44958 |
| K84A071 | South Korea | Temperate | 1984 | Mosquito | GIII | FJ938224 |
| JE-85 | Korea | Temperate | 1985 | Mosquito | GIII | GQ415350 |
| CN80 | Miaoli County, Taiwan | Temperate | 1985 | Mosquito | GIII | U44962 |
| JaOArS4385 | Oita Prefecture, Japan | Temperate | 1985 | Unknown | GIII | AB028258 |
| JaOArS5485 | Oita Prefecture, Japan | Temperate | 1985 | Unknown | GIII | AB028260 |
| JaOArS7485 | Oita Prefecture, Japan | Temperate | 1985 | Unknown | GIII | AB028259 |
| NT109 | Tiachung County, Taiwan | Temperate | 1985 | Mosquito | GIII | U44967 |
| NT113 | Tiachung County, Taiwan | Temperate | 1985 | Mosquito | GIII | U44968 |
| CH109 | Changhua County, Taiwan | Temperate | 1986 | Mosquito | GIII | U44959 |
| JE-86 | Korea | Temperate | 1986 | Mosquito | GIII | GQ415351 |
| JaOArK3786 | Oita Prefecture, Japan | Temperate | 1986 | Unknown | GIII | AB028261 |
| JaOArK7286 | Oita Prefecture, Japan | Temperate | 1986 | Unknown | GIII | AB028263 |
| JaOArS1186 | Oita Prefecture, Japan | Temperate | 1986 | Unknown | GIII | AB028262 |
| VN206 | Vietnam | Tropical | 1986 | Human | GIII | AY376460 |
| VN207 | Vietnam | Tropical | 1986 | Human | GIII | AY376461 |
| JKT27-085 | Central Java, Indonesia | Tropical | 1987 | Mosquito | GIII | JQ429307 |
| JKT27-087 | Central Java, Indonesia | Tropical | 1987 | Mosquito | GIII | JQ429308 |
| CH392 | Changhua County, Taiwan | Temperate | 1987 | Mosquito | GIII | U44961 |
| JE-87 | Korea | Temperate | 1987 | Mosquito | GIII | GQ415352 |
| K87P39 | Korea | Temperate | 1987 | Mosquito | GIII | U34927 |
| SH-3 | Shanghai, China | Temperate | 1987 | Human | GIII | AY243836 |
| K87A07 | South Korea | Temperate | 1987 | Mosquito | GIII | FJ938225 |
| K87A071 | South Korea | Temperate | 1987 | Mosquito | GIII | FJ938226 |
| JE-88 | Korea | Temperate | 1988 | Mosquito | GIII | GQ415353 |
| JaOArK6688 | Oita Prefecture, Japan | Temperate | 1988 | Unknown | GIII | AB028264 |
| K88A07 | South Korea | Temperate | 1988 | Mosquito | GIII | FJ938227 |
| K88A071 | South Korea | Temperate | 1988 | Mosquito | GIII | FJ938228 |
| 89P131 | Japan | Temperate | 1989 | Swine | GIII | FJ943467 |
| 89P141 | Japan | Temperate | 1989 | Swine | GIII | FJ943468 |
| 89P149 | Japan | Temperate | 1989 | Swine | GIII | FJ943469 |
| 89P160 | Japan | Temperate | 1989 | Swine | GIII | FJ943470 |
| 89P49 | Japan | Temperate | 1989 | Swine | GIII | FJ943466 |
| JE-89 | Korea | Temperate | 1989 | Mosquito | GIII | GQ415354 |
| JaOArK5789 | Oita Prefecture, Japan | Temperate | 1989 | Unknown | GIII | AB028265 |
| JaOArK6289 | Oita Prefecture, Japan | Temperate | 1989 | Unknown | GIII | AB028266 |
| K89A07 | South Korea | Temperate | 1989 | Mosquito | GIII | FJ938229 |
| VN49 | Vietnam | Tropical | 1989 | Human | GIII | AY376462 |
| VN50 | Vietnam | Tropical | 1989 | Human | GIII | AY376463 |
| CH1392 | Changhua County, Taiwan | Temperate | 1990 | Mosquito | GIII | U44960 |
| JaNAr0290 | Nagasaki Prefecture, Japan | Temperate | 1990 | Mosquito | GIII | AY427794 |
| JaNAr0590 | Nagasaki Prefecture, Japan | Temperate | 1990 | Mosquito | GIII | AY427795 |
| JaNAr0690 | Nagasaki Prefecture, Japan | Temperate | 1990 | Mosquito | GIII | AY427796 |
| JaNAr0990 | Nagasaki Prefecture, Japan | Temperate | 1990 | Mosquito | GIII | AY427797 |
| JaOArK3990 | Oita Prefecture, Japan | Temperate | 1990 | Unknown | GIII | AB028267 |
| JaOArK5990 | Oita Prefecture, Japan | Temperate | 1990 | Unknown | GIII | AB028268 |
| CH1949 | Changhua, Taiwan | Temperate | 1992 | Unknown | GIII | AF030549 |
| CH2195 | Changhua, Taiwan | Temperate | 1994 | Unknown | GIII | AF030550 |
| K94A071 | South Korea | Temperate | 1994 | Mosquito | GIII | FJ938217 |
| T1P1 | Liu Chi Islet, Taiwan | Tropical | 1997 | Mosquito | GIII | AF254453 |
| YN03-A151 | Yunnan, China | Temperate | 1998 | Mosquito | GIII | DQ404136 |
| 14178 | Lakhimpur, India | Temperate | 2001 | Human | GIII | EF623987 |
| 02-41 | Fujian, China | Temperate | 2002 | Human | GIII | AY555763 |
| 02-43 | Fujian, China | Temperate | 2002 | Human | GIII | AY555764 |
| 02-76 | Fujian, China | Temperate | 2002 | Human | GIII | AY555765 |
| 02-84 | Fujian, China | Temperate | 2002 | Human | GIII | AY555766 |
| 02-29 | Fujian, China | Temperate | 2002 | Human | GIII | AY555762 |
| 02-102 | Fujian, China | Temperate | 2002 | Human | GIII | AY555767 |
| HLJ02-134 | Heilongjiang, China | Temperate | 2002 | Midge | GIII | DQ404081 |
| HLJ02-136 | Heilongjiang, China | Temperate | 2002 | Midge | GIII | DQ404082 |
| HLJ02-144 | Heilongjiang, China | Temperate | 2002 | Mosquito | GIII | DQ404083 |
| HLJ02-170 | Heilongjiang, China | Temperate | 2002 | Mosquito | GIII | DQ404084 |
| 04940-4 | Maharashtra, India | Tropical | 2002 | Mosquito | GIII | EF623989 |
| TN207 | Taiwan | Unknown | 2002 | Mosquito | GIII | EU683895 |
| FJ03-31 | Fujian, China | Temperate | 2003 | Human | GIII | DQ404117 |
| FJ03-35 | Fujian, China | Temperate | 2003 | Human | GIII | DQ404118 |
| FJ03-39 | Fujian, China | Temperate | 2003 | Human | GIII | DQ404119 |
| FJ03-46 | Fujian, China | Temperate | 2003 | Human | GIII | DQ404120 |
| FJ03-56 | Fujian, China | Temperate | 2003 | Human | GIII | DQ404121 |
| FJ03-66 | Fujian, China | Temperate | 2003 | Human | GIII | DQ404122 |
| FJ03-67 | Fujian, China | Temperate | 2003 | Human | GIII | DQ404123 |
| FJ03-68 | Fujian, China | Temperate | 2003 | Human | GIII | DQ404124 |
| FJ03-69 | Fujian, China | Temperate | 2003 | Human | GIII | DQ404125 |
| FJ03-94 | Fujian, China | Temperate | 2003 | Human | GIII | DQ404126 |
| FJ03-97 | Fujian, China | Temperate | 2003 | Human | GIII | DQ404127 |
| GZ04-2 | Guizhou, China | Temperate | 2004 | Mosquito | GIII | DQ404109 |
| GZ04-4 | Guizhou, China | Temperate | 2004 | Mosquito | GIII | DQ404110 |
| GZ04-43 | Guizhou, China | Temperate | 2004 | Mosquito | GIII | DQ404113 |
| GZ04-71 | Guizhou, China | Temperate | 2004 | Mosquito | GIII | DQ404114 |
| GZ04-89 | Guizhou, China | Temperate | 2004 | Mosquito | GIII | DQ404115 |
| SH04-10 | Shanghai, China | Temperate | 2004 | Mosquito | GIII | DQ404107 |
| SH04-3 | Shanghai, China | Temperate | 2004 | Mosquito | GIII | DQ404105 |
| SH04-5 | Shanghai, China | Temperate | 2004 | Mosquito | GIII | DQ404106 |
| 04VN75 | Vietnam | Tropical | 2004 | Human | GIII | HQ009263 |
| 04VN79 | Vietnam | Tropical | 2004 | Human | GIII | HQ009264 |
| YNDL04-1 | Yunnan, China | Temperate | 2004 | Mosquito | GIII | DQ404137 |
| YNDL04-29 | Yunnan, China | Temperate | 2004 | Mosquito | GIII | DQ404139 |
| YNDL04-31 | Yunnan, China | Temperate | 2004 | Mosquito | GIII | DQ404140 |
| YNDL04-37 | Yunnan, China | Temperate | 2004 | Mosquito | GIII | DQ404141 |
| YNDL04-39 | Yunnan, China | Temperate | 2004 | Mosquito | GIII | DQ404142 |
| YNDL04-42 | Yunnan, China | Temperate | 2004 | Mosquito | GIII | DQ404143 |
| YNDL04-44 | Yunnan, China | Temperate | 2004 | Mosquito | GIII | DQ404144 |
| YNDL04-45 | Yunnan, China | Temperate | 2004 | Mosquito | GIII | DQ404145 |
| YNDL04-6 | Yunnan, China | Temperate | 2004 | Mosquito | GIII | DQ404138 |
| YNJH04-25-3 | Yunnan, China | Temperate | 2004 | Mosquito | GIII | DQ404148 |
| FJ05-139 | Fujian, China | Temperate | 2005 | Human | GIII | GQ856661 |
| FJ05-62 | Fujian, China | Temperate | 2005 | Human | GIII | GQ856660 |
| 57434 | Gorakhpur, India | Temperate | 2005 | Human | GIII | EF688625 |
| HL0505a | Hualien County, Taiwan | Temperate | 2005 | Mosquito | GIII | GQ260610 |
| HL0506a | Hualien County, Taiwan | Temperate | 2005 | Mosquito | GIII | GQ260615 |
| KH0505a | Kaohsiung County, Taiwan | Tropical | 2005 | Mosquito | GIII | GQ260608 |
| KH0505b | Kaohsiung County, Taiwan | Tropical | 2005 | Mosquito | GIII | GQ260609 |
| TP0506a | Taipei County, Taiwan | Temperate | 2005 | Mosquito | GIII | GQ260616 |
| TC0506a | Tiachung County, Taiwan | Temperate | 2005 | Mosquito | GIII | GQ260613 |
| TC0506b | Tiachung County, Taiwan | Temperate | 2005 | Mosquito | GIII | GQ260614 |
| GP05 | Uttar Pradesh, India | Temperate | 2005 | Human | GIII | FJ979830 |
| YL0506a | Yilan County, Taiwan | Temperate | 2005 | Mosquito | GIII | GQ260611 |
| YL0506b | Yilan County, Taiwan | Temperate | 2005 | Mosquito | GIII | GQ260612 |
| CSF-2522 | Taiwan | Unknown | 2006 | Human | GIII | GQ260621 |
| TC0605a | Tiachung County, Taiwan | Temperate | 2006 | Mosquito | GIII | GQ260618 |
| TC0605b | Tiachung County, Taiwan | Temperate | 2006 | Mosquito | GIII | GQ260619 |
| YL0605a | Yilan County, Taiwan | Temperate | 2006 | Mosquito | GIII | GQ260617 |
| YL0606a | Yilan County, Taiwan | Temperate | 2006 | Mosquito | GIII | GQ260620 |
| CH0706a | Changhua County, Taiwan | Temperate | 2007 | Swine | GIII | GQ260624 |
| FJ07-51 | Fujian, China | Temperate | 2007 | Human | GIII | GQ856662 |
| HL0706a | Hualien County, Taiwan | Temperate | 2007 | Mosquito | GIII | GQ260625 |
| TN0705a | Tainan County, Taiwan | Tropical | 2007 | Swine | GIII | GQ260623 |
| TPC0706b | Taipei City, Taiwan | Temperate | 2007 | Mosquito | GIII | GQ260627 |
| TY0704a | Taoyuan County, Taiwan | Temperate | 2007 | Swine | GIII | GQ260622 |
| TPC0706a | Taipei City, Taiwan | Temperate | 2007 | Mosquito | GIII | GQ260626 |
| Mo/Chongmingdao/12/2008 | Chongmingdao, China | Temperate | 2008 | Mosquito | GIII | GU253955 |
| Mo/Deqing/11/2008 | Deqing, China | Temperate | 2008 | Mosquito | GIII | GU253961 |
| FJ08-48 | Fujian, China | Temperate | 2008 | Human | GIII | GQ856663 |
| FJ08-65 | Fujian, China | Temperate | 2008 | Human | GIII | GQ856664 |
| HLJ08-01 | Heilongjiang, China | Temperate | 2008 | Swine | GIII | GQ495004 |
| HLJ08-02 | Heilongjiang, China | Temperate | 2008 | Swine | GIII | GQ495005 |
| HL0805a | Hualien, Taiwan | Temperate | 2008 | Mosquito | GIII | GQ260628 |
| Mo/Jintan/9/2008 | Jintan, China | Temperate | 2008 | Mosquito | GIII | GU253954 |
| Mo/Nanjing/10/2008 | Nanjing, China | Temperate | 2008 | Mosquito | GIII | GU253951 |
| TC0806a | Taichung County, Taiwan | Temperate | 2008 | Mosquito | GIII | GQ260630 |
| TPC0806a | Taipei City, Taiwan | Temperate | 2008 | Mosquito | GIII | GQ260634 |
| YL0805a | Yilan County, Taiwan | Temperate | 2008 | Mosquito | GIII | GQ260629 |
| YL0806a | Yilan County, Taiwan | Temperate | 2008 | Mosquito | GIII | GQ260631 |
| YL0806e | Yilan County, Taiwan | Temperate | 2008 | Mosquito | GIII | GQ260632 |
| Mo/Haimen/3/2009 | Haimen, China | Temperate | 2009 | Mosquito | GIII | GU253947 |
| Mo/Haimen/6/2009 | Haimen, China | Temperate | 2009 | Mosquito | GIII | GU253950 |
| Mo/Qidong/4/2009 | Haimen, China | Temperate | 2009 | Mosquito | GIII | GU253948 |
| JEV/eq/Haryana/H225/2009 | Haryana, India | Temperate | 2009 | Equid | GIII | GQ387646 |
| JEV/eq/India/H225/2009 | Hisar, India | Temperate | 2009 | Equid | GIII | HQ018880 |
| Sw(blood)/Jintan/15/2009 | Jintan, China | Temperate | 2009 | Swine | GIII | GU253953 |
| Sw(sperm)/Ningbo/13/2009 | Ningbo, China | Temperate | 2009 | Swine | GIII | GU253956 |
| Mo/Qidong/5/2009 | Qidong, China | Temperate | 2009 | Mosquito | GIII | GU253949 |
| Sw(sperm)/Rizhao/8/2009 | Rizhao, China | Temperate | 2009 | Swine | GIII | GU253960 |
| Sw(blood)/Rugao/14/2009 | Rugao, China | Temperate | 2009 | Swine | GIII | GU253957 |
| JEV-NJ1 | Sichuan, China | Temperate | 2009 | Mosquito | GIII | HM234674 |
| Mo/Yixing/2/2009 | Yixing, China | Temperate | 2009 | Mosquito | GIII | GU253959 |
| Mo/Zhenjiang/1/2009 | Zhenjiang, China | Temperate | 2009 | Mosquito | GIII | GU253958 |
| JKT7089 | Bantul, Java, Indonesia | Tropical | 1981 | Mosquito | GIV | JQ429309 |
| JKT7180 | Central Java, Indonesia | Tropical | 1981 | Mosquito | GIV | JQ429310 |
| JKT6468 | Flores, Indonesia | Tropical | 1981 | Mosquito | GIV | AY184212 |
| Muar | Malaysia | Tropical | 1952 | Human | GV | HM596272 |
| XZ0934 | China | Temperate | 2009 | Mosquito | GV | JF915894 |
| 10-1827 | South Korea | Temperate | 2010 | Mosquito | GV | JN587258 |
