# Supplementary material for: Phylogeography of Japanese Encephalitis Virus: Genotype Is Associated with Climate
Source: PLoS Negl Trop Dis. 2013 Aug 29;7(8):e2411. doi: 10.1371/journal.pntd.0002411 (PMC3757071; doi:10.1371/journal.pntd.0002411)
Supplement: Table S3 — Analysis of JEV phylogeographic structure. (DOCX) [file pntd.0002411.s005.docx]

Table S3. Analysis of JEV phylogeographic structure.

| **Statistic** | **Observed mean (95% CI)** | **Null mean (95% CI)** | **p-value** |
| --- | --- | --- | --- |
| AI | 1.52 (1.13, 1.91) | 13.38 (11.88, 14.83) | 0.00 |
| PS | 29.35 (26.00, 33.00) | 105.79 (101.46, 109.49) | 0.00 |
| UniFrac | 0.67 (0.59, 0.73) | 0.36 (0.26, 0.44) | 1.00 |
| NT | 2146.76 (1768.35, 2692.94) | 2684.00 (2440.40, 2965.32) | 0.00 |
| NR | 9690017.00 (6703190.50, 14393071.00) | 10267356.00 (10150377.00, 10351031.00) | 0.00 |
| PD | 5696.88 (4652.83, 7221.35) | 6556.90 (6314.05, 6824.98) | 0.00 |
| MC, Temperate | 85.97 (86.00, 86.00) | 16.12 (11.21, 25.01) | < 0.00 |
| MC, Tropical | 25.97 (26.00, 26.00) | 2.27 (1.95, 3.01) | < 0.00 |
