# Supplementary material for: Phylogeography of Japanese Encephalitis Virus: Genotype Is Associated with Climate
Source: PLoS Negl Trop Dis. 2013 Aug 29;7(8):e2411. doi: 10.1371/journal.pntd.0002411 (PMC3757071; doi:10.1371/journal.pntd.0002411)
Supplement: Table S4 — Genotype-defining nonsynonymous substitutions within the E protein. (DOCX) [file pntd.0002411.s006.docx]

**Table S4.** Genotype-defining nonsynonymous substitutions within the E protein.

| **Domain^1^** | **Site** | **GV** | **GIV** | **GIII** | **GII** | **GI** | **GI-a** | **GI-b** | **Type** |
| --- | --- | --- | --- | --- | --- | --- | --- | --- | --- |
| I | 15 | V | A | A | A | A | A | A | Conservative |
|  | 38 | K | R | K | K | K | K | K | Conservative |
|  | 51 | T | S | S | S | S | S | S | Conservative |
|  | 52 | E | Q | Q | Q | Q | Q | Q | Non-conservative |
| II | 58 | T | S | S | S | S | S | S | Conservative |
|  | 64 | T | S | S | S | S | S | S | Conservative |
|  | 66 | A | T | T | T | T | T | T | Non-conservative |
|  | 83 | T | E | E | E | E | E | E | Non-conservative |
|  | 91 | Y | F | F | F | F | F | F | Conservative |
|  | 120 | V | S | S | S | S | S | S | Non-conservative |
| I | 122 | S | T | T | T | T | T | T | Conservative |
|  | 123 | H | S | S | S | S | S | S | Non-conservative |
|  | 128 | K | R | R | R | R | R | R | Conservative |
|  | 129 | I | T | T | T | M | M | M | Non-conservative |
|  | 141 | V | I | I | I | I | I | V | Conservative |
|  | 149 | A | S | S | S | S | S | S | Non-conservative |
|  | 156 | T | T | S | S | S | S | S | Conservative |
|  | 159 | I | V | V | V | V | V | V | Conservative |
|  | 169 | I | I | V | V | V | V | V | Conservative |
|  | 188 | M | L | L | L | L | L | L | Conservative |
|  | 196 | F | L | L | L | L | L | L | Non-conservative |
|  | 204 | L | M | M | M | M | M | M | Conservative |
|  | 219 | N | H | H | H | H | H | H | Non-conservative |
|  | 222 | S | A | A | S | S | S | S | Non-conservative |
| II | 226 | L | T | T | T | T | T | T | Non-conservative |
|  | 232 | N | A | A | A | A | A | A | Non-conservative |
|  | 238 | I | L | L | L | L | L | L | Conservative |
|  | 261 | A | G | G | G | G | G | G | Non-conservative |
| III | 311 | S | A | A | A | A | A | A | Non-conservative |
|  | 327 | Q | L | S | T | T | T | T | Non-conservative/Conservative |
|  | 329 | T | S | S | S | S | S | S | Conservative |
|  | 331 | T | S | S | S | S | S | S | Conservative |
|  | 340 | S | V | V | V | V | V | V | Non-conservative |
|  | 348 | L | M | M | M | M | M | M | Conservative |
|  | 365 | T | S | S | S | S | S | S | Conservative |
|  | 366 | A | S | A | S | S | S | S | Non-conservative |
|  | 374 | L | M | M | M | M | M | M | Conservative |
|  | 382 | F | Y | Y | Y | Y | Y | Y | Conservative |
|  | 402 | S | T | T | T | T | T | T | Conservative |
| Stem-anchor | 408 | T | S | S | S | S | S | S | Conservative |
|  | 466 | A | V | A | A | A | A | A | Conservative |
|  | 473 | I | I | V | V | V | V | V | Conservative |
|  | 482 | L | M | L | L | L | L | L | Conservative |
|  | 492 | L | V | V | V | V | V | V | Conservative |

^1^The E protein is comprised of a dimer with each monomer having three noncontiguous structurally distinct domains: domain I is centrally located and acts as a hinge between the other two domains, domain II is the dimerization domain and contains the fusion peptide at its distal end and domain III is the receptor-binding domain (Rey et al., 1995). The stem-anchor region, located at the carboxy-terminal of the E protein, plays a critical role in membrane fusion and virus assembly (Allison *et al*., 1999).
