# Supplementary material for: Phylogeography of Japanese Encephalitis Virus: Genotype Is Associated with Climate
Source: PLoS Negl Trop Dis. 2013 Aug 29;7(8):e2411. doi: 10.1371/journal.pntd.0002411 (PMC3757071; doi:10.1371/journal.pntd.0002411)
Supplement: Table S5 — DEPS analysis of the JEV E protein alignment. (DOCX) [file pntd.0002411.s007.docx]

**Table S5.** DEPS analysis of the JEV E protein alignment.

| **Residue** | **p-value** | **Bias** | **Proportion (%)** |
| --- | --- | --- | --- |
| R | < 0.00 | 11.9 | 3.4 |
| M | < 0.00 | 43.0 | 2.0 |
| G | < 0.00 | 36.5 | 0.5 |
| N | < 0.00 | 23.7 | 0.6 |
| K | < 0.00 | 22.2 | 2.3 |
| P | < 0.00 | 21.8 | 2.0 |
| Y | < 0.00 | 16.7 | 4.8 |
