# Supplementary material for: Phylogeography of Japanese Encephalitis Virus: Genotype Is Associated with Climate
Source: PLoS Negl Trop Dis. 2013 Aug 29;7(8):e2411. doi: 10.1371/journal.pntd.0002411 (PMC3757071; doi:10.1371/journal.pntd.0002411)
Supplement: Table S6 — Amino acid sites within the E protein of JEV identified by the DEPS analyses to be under directional selection. (DOCX) [file pntd.0002411.s008.docx]

**Table S6.** Amino acid sites within the E protein of JEV identified by the DEPS analyses to be under directional selection.

| **Site** | **BF** | **Preferred residue** | **Inferred substitutions^1^** |
| --- | --- | --- | --- |
| 76 | 1.32 x 10^8^ | M | A_0_↔_2_T, M_0_↔_6_T |
| 83 | 7975.75 | K | A_0_↔_1_E, D_0_↔_2_E, E_1_↔_0_G, E_4_↔_0_K, E_1_↔_0_Q, E_0_↔_1_T |
| 84 | 1236.76 | K | G_35_↔_11_K, G_2_↔_0_R |
| 87 | 3070.10 | Y | D_11_↔_35_G, D_1_↔_0_Y, G_2_↔_0_Y |
| 123 | 8.57 x 10^7^ | N and R | H_1_↔_0_S, K_0_↔_1_N, N_0_↔_9_S, R_0_↔_6_S |
| 129 | 117.52 | M | I_1_↔_2_T, M_0_↔_2_T |
| 138 | 7495.68 | K | E_4_↔_0_K, E_1_↔_0_Q |
| 176 | 102.08 | R | I_2_↔_O_R, I_3_↔_1_T, I_2_↔_0_V |
| 209 | 69197.00 | R | E_0_↔_1_K, K_8_↔_0_R |
| 227 | 2.78 x 10^6^ | P | P_0_↔_6_S |
| 240 | 414.40 | M | G_1_↔_0_L, G_36_↔_11_M |
| 306 | 1.26 x 10^8^ | G | E_7_↔_0_G, E_2_↔_0_K |
| 397 | 6021.03 | Y | H_1_↔_0_P, H_5_↔_0_Y |

^1^Amino acid substitutions inferred when A_b_↔_c_D indicates b substitutions from A to D and c substitutions from D to A.
